# Evaluation of Treatment Patterns and Maintenance Dose Titration Among Patients With Crohn’s Disease Initiating Biologics With 3 Years of Follow-Up

**DOI:** 10.36469/001c.88947

**Published:** 2023-11-20

**Authors:** Ruizhi Zhao, Zhijie Ding, Parul Gupta, Laurence Gozalo, Robert Bruette, Victor M. Johnson, Keshia Maughn, Yihang Liu, Sumesh Kachroo

**Affiliations:** 1 Janssen Scientific Affairs, LLC, Horsham, Pennsylvania; 2 STATinMED, LLC, Dallas, Texas

**Keywords:** Crohn’s disease, dose titration, persistence, adalimumab, certolizumab pegol, infliximab, biosimilar, ustekinumab, vedolizumab

## Abstract

**Background:** There is limited real-world evidence on treatment patterns of patients with Crohn’s disease (CD) initiating biologics with an extensive follow-up period. This study describes persistence and dose titration among CD patients with 3 years of follow-up.

**Methods:** This retrospective observational study was conducted using the STATinMED RWD Insights all-payer medical and pharmacy data. Adult patients with at least 1 CD medical claim and at least 1 medical/pharmacy claim for a biologic (adalimumab [ADA], certolizumab pegol (CZP), infliximab [IFX] and its biosimilar products [IFX-BS], ustekinumab [UST], and vedolizumab [VDZ]) between September 2016 and October 2018 were identified. Commercially insured patients with continuous capture for at least 12 months before and at least 36 months after biologics initiation were selected. Confirmed CD patients were included in the final cohort. Baseline patient characteristics and treatment patterns over the 3-year follow-up period were evaluated. Results were summarized using means and SD or counts and percentages.

**Results:** A total of 2309 confirmed patients with CD were identified (847 [36.7%] IFX, 534 [23.1%] ADA, 486 [21.1%] VDZ, 394 [17.1%] UST, 85 [3.7%] CZP, and 72 [3.1%] IFX-BS). CZP and IFX-BS were excluded due to small sample sizes. Approximately half of CD patients were between ages 35 and 54. Patients on UST had a higher Charlson Comorbidity Index score. Common comorbidities (>10%) included anemia, anxiety, depression, and hypertension. Persistence over 3 years’ follow-up was highest for UST (61.4%) patients, followed by VDZ (58.0% ), ADA (52.1% , and IFX (48.1%). The discontinuation rate without switch or restart was highest for ADA (37.3%), followed by UST (30.7%), IFX (28.1%), and VDZ (25.3%). Over the 3 years of follow-up, the dose titration rate was highest for IFX (76.5%) and lowest for UST (50.8%). In particular, UST had the lowest dose escalation rate (35.5%) and highest dose-reduction rate (16.5%).

**Conclusions:** Patients with CD on UST had the highest persistence and lowest dose escalation across different biologic users over the 3-year follow-up period, possibly suggesting a better clinical response of UST. Future studies with longer follow-up adjusting for confounders are needed to better understand treatment patterns among biologics users.

## INTRODUCTION

Crohn’s disease (CD) is a primary type of inflammatory bowel disease (IBD). It is a relapsing chronic inflammatory disorder of the gastrointestinal tract and can involve any part of the gastrointestinal tract from the mouth to the anus.^1,2^ Presenting symptoms may include abdominal pain, diarrhea, fatigue, weight loss, failure to grow, anemia, or recurrent fistulas, although variability has been noted.^1,3^ Stricturing or penetrating complications are also prevalent in patients with CD and often result in hospitalization and surgical intervention.^4^ Estimates indicate that approximately 780 000 Americans had CD in 2014, accompanied by an increasing prevalence.^5^

A variety of therapies are available to manage CD in the US adult population. For moderate to severe CD, conventional treatments include higher-dose oral or intravenous corticosteroids and immunomodulators (azathioprine, mercaptopurine, methotrexate, cyclosporine).^6^ However, studies have shown that these treatments trigger no response in approximately 20% to 40% of patients.^7,8^ Clinical guidelines recommend treatment with biologic agents for those who do not respond, lose response, or are intolerant of conventional treatments.^9^ In the United States, biologic therapies approved for CD treatment include infliximab (IFX; Remicade®), adalimumab (ADA; Humira®),^10^ certolizumab pegol (CZP; Cimzia®), vedolizumab^11^ (VDZ; Entyvio®), and natalizumab (NTZ; Tysabri®). In September 2016, the US Food and Drug Administration (FDA) approved ustekinumab (UST, Stelara®), an additional biologic therapy to treat CD. Specifically, UST was approved to treat patients who failed conventional therapy, prior therapy, or multiple anti–tumor necrosis factor (TNF) agents.^12–14^ A randomized clinical trial that evaluated UST treatment effect on biologic-experienced moderate-to-severe refractory patients with CD reported increased rates of response to induction and maintenance doses with the drug.^15^ Moreover, the FDA approved risankizumab (Skyrizi®) in 2022 as the first and only specific interleukin-23 inhibitor for the treatment of adults with moderately to severely active CD.

Variations in tolerance and response to treatment have been noted with biologics and can inform treatment sequence selection and guidelines, resulting in dose adjustments, augmentation, and therapy changes and discontinuation among patients with CD.^16^ In a real-world study examining TNF-α inhibitors among patients with IBD, persistence was only 64.2% for ADA and 56.5% for IFX among patients with CD during the first year after initiation.^17^ A study by Rubin et al calculated IBD-related dose escalation rates for ADA, CZP, and for IFX to be between 17% and 35%.^18^ Another study reported CD-related dose titration rates of 32% to 38% for anti-TNF agents like ADA, CZP, and IFX.^19^ This study, however, did not report the rates associated with individual biologics.

Limited literature is available related to real-world evidence (RWE) on long-term treatment patterns and dose titration among patients with CD using biologics. A recently published study evaluated real-world treatment patterns among patients with CD prescribed with UST, VDZ, ADA, IFX, or CZP using pooled administrative commercial claims data with 1-year follow-up.^20^ Between 64.9% and 87.2% of bio-naïve and 60.7% to 86.3% of biologic-experienced patients were persistent over the 1-year follow-up period. Despite the 1-year data, there remains a need to further explore long-term treatment patterns and dose titration among patients with CD treated with biologics. We aimed to descriptively evaluate persistence, dose titration, and other treatment patterns among patients with CD who were prescribed biologics over a 3-year follow-up period using STATinMED’s licensed Real-World Data (RWD) Insights, an all-payer administrative claims database.

## METHODS

### Data Source

Data for this study were obtained from STATinMED RWD Insights, an all-payer medical and pharmacy claims database. RWD Insights is a large-scale statewide database that systematically aggregates medical claims and pharmacy claims from a variety of payer sources.^21^ The source provides insight to approximately 80% of the US healthcare system, encompassing numerous payers sourced directly from claims clearinghouses, which are responsible for managing claims transactions between payers and providers across the US states. These data offer comprehensive insight for claims at the patient level across the US healthcare system and in-depth visibility into payer account details. RWD Insights data contain both private and government healthcare insurance entities including commercial, Medicare Fee-for-Service, Medicare Advantage, and Medicaid in all 50 states since 2014. This unique data feature allows longitudinal tracking of patients in the US healthcare system as they change insurance carriers and plans over time. Comprehensive coverage information makes it possible to understand the switch patterns in a longer follow-up period across multiple providers and provides insight into biologic treatment patterns among patients with CD in the United States.

Both medical and pharmacy claims for the commercial population from RWD Insights were used for this study. The medical claims were coded using the *International Classification of Disease (ICD), Ninth Revision, Clinical Modification* (ICD-9-CM), ICD-10-CM (implemented October 1, 2015), or Current Procedural Terminology (CPT), whereas the pharmacy claims were coded using National Drug Code (NDC) or Health Care Common Procedure Coding Systems (HCPCS). All data were de-identified, tokenized, and HIPAA compliant.

### Study Design and Patient Selection

**Figure 1.**
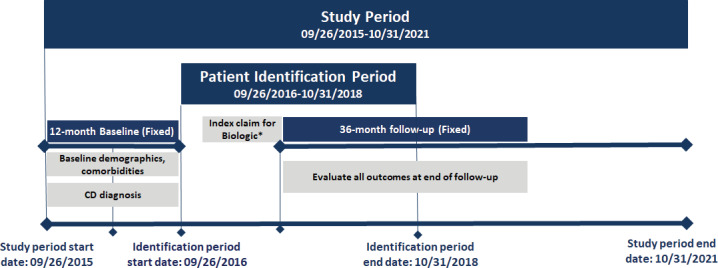
General Study Design The illustrated study design is for illustration purposes only, and the respective baseline and follow-up periods may not be to scale. *This study categorized the patients into multiple cohorts based on all the biologics they used during the identification period. Index date is the date of the first medical or pharmacy prescription for the biologic observed during the identification period. Abbreviation: CD, Crohn’s disease.

**Figure 2.**
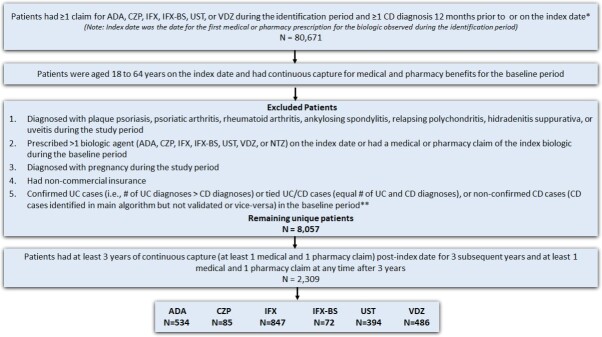
Patient Selection Criteria *Patients could be assigned to multiple cohorts based on the biologics used during the identification period. **A modified algorithm based on the Manitoba study was used to exclude patients with a UC diagnosis or those with an equal number of UC and CD diagnoses.^21–23^ Abbreviations: ADA, adalimumab; CD, Crohn’s disease; CZP, certolizumab pegol; IFX, infliximab; IFX-BS, infliximab biosimilar; NTZ, natalizumab; UC; ulcerative colitis; UST, ustekinumab; VDZ, vedolizumab.

This is a longitudinal retrospective observational study. The study design is shown in **Figure 1**, and the patient selection criteria are described in **Figure 2**.

Adult patients (18-64 years) with at least 1 medical or pharmacy commercial claim for UST, VDZ, ADA, IFX, IFX-biosimilar (IFX-BS enlisted in **Appendix 1.3**), or CZP between September 26, 2016, and October 31, 2018, were identified. Patients on risankizumab (Skyrizi) were not included in this study because this drug was not approved for CD patients during the study period. All biologics were identified using HCPCS codes or NDC codes (see **Appendix Market Definition 1.2-1.5**). The index date was defined as the date of the first medical or pharmacy claim for any biologic (“index biologic”) treatment during the identification period. Patients were assigned to multiple cohorts based on all the biologics they used during the identification period. Patients were required to have at least 1 CD diagnosis (see **Appendix Market Definition 1.1**) during the 12-month baseline period. In addition, they were required to have continuous capture of a commercial health plan with both medical and pharmacy benefits for the 12-month baseline and 36-month follow-up periods.

Patients were excluded from the study if they: (1) had a diagnosis code (ICD-9-CM or ICD-10-CM) for plaque psoriasis, psoriatic arthritis, rheumatoid arthritis, ankylosing spondylitis, relapsing polychondritis, hidradenitis suppurativa, or uveitis during the study period (September 26, 2015–October 31, 2021); (2) had a diagnostic code for pregnancy during the study period; (3) had more than 1 biologic agent prescription (UST, VDZ, ADA, IFX, IFX biosimilar, CZP, or NTZ) on the index date; and (4) had a medical or pharmacy claim of the index drug during the 12 months before the index baseline period. Lastly, a modified algorithm based on the Manitoba study was used to further exclude patients with an ulcerative colitis (UC) diagnosis or those with equal number of UC and CD diagnoses (**Figure 2**) in the 12-month baseline period.^21^ Patients taking NTZ on the index date were excluded. This was consistent with the Cochrane IBD Group’s recommendation, which suggests that NTZ is rarely used in patients unresponsive to current medical therapies and is associated with serious safety concerns due to its link with the development of progressive multifocal leukoencephalopathy, which could lead to patient death.^24^ Furthermore, patients receiving CZP and IFX-BS were not included in the analysis due to low sample size.

### Study Measures

Patient demographic characteristics were examined, including age on the index date, sex, US geographic region (**Appendix 1.7**), and clinical characteristics including baseline Charlson Comorbidity Index (CCI) score and individual comorbidities (**Appendix 1.8**). Individual comorbidities were identified using the diagnosis codes during the baseline period (**Table 1**) and included anemia, anxiety, atherosclerosis, celiac disease, cholelithiasis, chronic pain, depression, diabetes, fatigue, fistula, hyperlipidemia, hypertension, obesity, and venous thromboembolism. Low numbers (<5) were not reported in **Table 1**. During the follow-up period, treatment patterns including persistence, discontinuation, medication adherence, and dose titration were evaluated.

**Table 1.** Baseline Characteristics Among CD Patients Using Biologics With 3 Years of Follow-Up

**Characteristics**	**Biologics**							
	**UST (N = 394)**		**VDZ (N = 486)**		**IFX (N = 847)**		**ADA (N = 534)**	
	**N/Mean**	**%/SD**	**N/Mean**	**%/SD**	**N/Mean**	**%/SD**	**N/Mean**	**%/SD**
Age on index date, y								
Mean	44.0	12.8	45.3	12.0	43.7	13.3	43.5	12.8
Age group								
18-34	101	25.6%	97	20.0%	224	26.4%	128	24.0%
35-54	191	48.5%	254	52.3%	400	47.2%	280	52.4%
55-64	102	25.9%	135	27.8%	223	26.3%	126	23.6%
Sex								
Male	184	46.7%	212	43.6%	356	42.0%	244	45.7%
Female	210	53.3%	274	56.4%	491	58.0%	290	54.3%
US geographic region								
Northeast	69	17.5%	95	19.5%	161	19.0%	75	14.0%
North Central	147	37.3%	171	35.2%	280	33.1%	177	33.1%
South	120	30.5%	153	31.5%	260	30.7%	201	37.6%
West	58	14.7%	67	13.8%	146	17.2%	81	15.2%
Index year								
2016	31	7.9%	65	13.4%	112	13.2%	76	14.2%
2017	209	53.0%	250	51.4%	467	55.1%	303	56.7%
2018	154	39.1%	171	35.2%	268	31.6%	155	29.0%
CCI score	0.75	2.00	0.59	1.67	0.47	1.43	0.54	1.64
Comorbidities								
Anemia	119	30.2%	102	21.0%	143	16.9%	93	17.4%
Anxiety	78	19.8%	85	17.5%	108	12.8%	75	14.0%
Celiac disease	24	6.1%	14	2.9%	18	2.1%	6	1.1%
Chronic pain	35	8.9%	40	8.2%	40	4.7%	31	5.8%
Depression	72	18.3%	84	17.3%	105	12.4%	76	14.2%
Diabetes	29	7.4%	35	7.2%	53	6.3%	33	6.2%
Fatigue	56	14.2%	55	11.3%	73	8.6%	41	7.7%
Fistula	25	6.3%	29	6.0%	57	6.7%	18	3.4%
Hyperlipidemia	38	9.6%	52	10.7%	79	9.3%	49	9.2%
Hypertension	80	20.3%	85	17.5%	133	15.7%	101	18.9%
Obesity	46	11.7%	48	9.9%	80	9.4%	54	10.1%
Venous thromboembolism	11	2.8%	8	1.6%	13	1.5%	4	0.8%

Due to rounding of the percentages, percentages may not add to 100%.

Abbreviations: ADA, adalimumab; IFX, infliximab; UST, ustekinumab; VDZ, vedolizumab.

Persistence was defined as the proportion of patients who remained on the index biologic without an allowable gap (gap of approximately double the maintenance dosing interval) of more than 60 days for ADA and more than 120 days for IFX, UST, and VDZ between the run-out date of 2 consecutive biologic claims. The approach of 2 times the maintenance dosing interval is based on past literature on similar studies because these patients typically receive 2 maintenance doses per claim.^20,25^ Patients who were not persistent with their index biologic were classified as discontinuers.

Patients who were administered a non-index biologic during follow-up were considered switchers. The switch may have occurred before (defined as switch within allowable gap) or after the allowable gap (defined as switch after the allowable gap). Patients who restarted their index biologic after the allowable gap during follow-up were defined as restarters. The study also examined patients who were not administered/prescribed any biologics after the discontinuation date (defined as the run-out date of the last index medication claim or the switch date, whichever occurred first) without any restart or switch. These patients were defined as discontinued without restart or switch of any therapy and were considered true discontinuers.

Medication adherence was evaluated by assessing the medication possession ratio (MPR) and proportion of days covered (PDC). The MPR was defined as the sum of the days of supply of biologic (identified from medical and pharmacy claims) before discontinuation or switch divided by the treatment duration days (MPR based on treatment duration) or follow-up days (MPR based on follow-up period). The overall treatment duration was defined as the number of days of uninterrupted treatment during follow-up from the index date to the switch date to a different biologic, the run-out date of last medication prior to a non-allowable gap, or end of follow-up, whichever was earliest. As for the total follow-up period, patient data were assessed until the end of the study period. The former definition offers an estimate of adherence in relation to the prescription period, while the latter definition estimates adherence in relation to the total follow-up period. Patients with MPR at least 0.80 were reported as adherent patients.

PDC was also calculated as an indicator for mediation adherence. This estimate is a more conservative method of adherence than MPR because it does not double-count any overlap between biologic administrations. It was defined as the sum of the days covered by the biologic (days of supply minus any overlapping days between administrations) before discontinuation or switch divided by either treatment duration (PDC based on treatment duration) or follow-up days (PDC based on follow-up period). Patients with a PDC of at least 80% were reported as adherent patients.

Dose titration was assessed among eligible patients, defined as patients who had maintenance therapy with at least 2 maintenance doses identified by the same type claims (medical claims or pharmacy claims) during the follow-up period. The start of the maintenance period was defined based on the FDA label–recommended visit schedules and days (with a 20% variation) after the index biologic: at least the fourth claim after at least 78 days for VDZ, IFX, and IFX-BS, at least the third claim after at least 22 days for ADA and CZP, and at least the second claim after at least 45 days for UST.

The average daily maintenance dose was also calculated; for medical claims, unit information and equivalent days of supply from clinical guidelines was used to calculate units per day. For pharmacy claims, average daily dose was calculated by Quantities × Strength ÷ Equivalent Days of Supply.

When using claims data, medical claims-related J-codes do not provide enough detail to determine drug strength. Moreover, the variations related to the payer and billing make dose titration a challenging step. To address the heterogeneity without making assumptions, unit information provided in the medical claims was used to measure dose calculation. For each maintenance refill of the same product under the same benefit (medical or pharmacy), the first instance of percentage change was calculated as the difference in current dose with respect to the average maintenance dose. This approach of calculating dose change avoids the need for assumptions about doses, and it is consistently applicable across different biologics. As a result, the findings were represented on a common scale of change in units, serving as a measure of dose titration, regardless of variations in medication units, formulations (IV or SubQ as for UST), or dependency on weight (as for IFX and its biosimilar products) for dose calculation. Dose titration was assessed during the maintenance phase and censored at the earliest of switch or end of study period.

Once maintenance therapy was initiated after the index drug, dose titration was calculated for each fill as either an unchanged, escalation, or reduction with respect to the initial biologic dose. We further categorized the dose reduction into a decrease of ≤50% or >50% from the starting average dose and an increase of <50%, 51%-100%, or >100% from the starting average dose as dose escalation.

There could be situations such as when a person had both escalation and a reduction during the follow-up period. For such situations, to create mutually exclusive groups, escalation was prioritized over reduction and unchanged for each patient and maintenance year. If a patient switched drugs after establishing a maintenance dose, they would not be captured in the subsequent years.

### Statistical Analysis

Descriptive analyses were performed to explore patient demographics and clinical characteristics at baseline and to examine treatment patterns associated with selective biologics during the follow-up period. Frequency and percentages for categorical variables and means with SD for continuous variables were reported. All analyses were conducted using SAS 9.4.

### Ethical Considerations

No identifiable patient information or medical records were disclosed for this study except in compliance with applicable law. Since the study did not involve the collection, use, or transmittal of individually identifiable data, Institutional Review Board (IRB) approval was not required. Both the data and the security of our offices where we kept the data met the Health Insurance Portability and Accountability Act (HIPAA) requirements.

## RESULTS

A total of 80 671 patients had at least 1 claim for the biologic of interest and one claim for CD diagnosis, with 33 425 of these meeting both the age on the index date and pre-index continuous capture criteria (**Figure 2**). Of these patients, 25 368 were excluded due to having other conditions/diseases, timing of biologic treatment, pregnancy, noncommercial insurance, or unconfirmed CD, leaving 8057 patients remaining. Further applying the continuous capture criteria for the follow-up period, 2309 patients with CD were identified as the final sample for the study. Approximately 36.7% of patients received IFX, followed by 23.1% on ADA, 21.2% on VDZ, 17.1% on UST, 3.7% on CZP, and 3.1% on IFX-BS.

### Baseline Characteristics

Baseline patient demographic and clinical characteristics by biologics are shown in **Table 1**. Demographic characteristics were similar across different biologic treatment groups. The mean age of patients on the index date ranged from 43.5 to 45.3 years, with the majority between 35 and 54 years (47.2%-52.4%). Female patients comprised slightly over half of the sample (53.3%-58.0%). Most patients with CD with biologic treatment (63.7%-70.8%) resided in the North Central or South US regions. In the 3-year identification period, 2016 had the least patients (<15%) initiating a biologic treatment. As expected, UST was the least used biologic (7.9%) in 2016, given that it was approved to the market that year. Common comorbidities among patients with CD included anemia (16.9%-30.2%), hypertension (15.7%-20.3%), anxiety (12.8%-19.8%), depression (12.4%-18.3%), fatigue (7.7%-14.2%), obesity (9.5%-11.7%), and hyperlipidemia (9.2%-10.7%).

Patients receiving UST had a somewhat higher mean CCI score (0.75 ± 2.00) than patients receiving VDZ (0.59 ±1.67), IFX (0.47 ± 1.43) or ADA (0.54 ± 1.64). The UST group generally had the highest proportion of patients with individual comorbidities compared with patients receiving other biologics (**Table 1**).

### Outcome Measures

**Persistence and discontinuation**: Based on a variable allowable gap period, persistence over 3 years’ follow-up was highest for UST patients (61.4%), followed by VDZ (58.0%), ADA (52.1%), and IFX (48.1%) (Table 2). The unadjusted overall discontinuation rate was highest for IFX (52.0%), followed by ADA (47.9%), VDZ (42.0%), and UST (38.6%). Discontinuation without switch or restart was highest for ADA (37.3%), followed by UST (30.7%), IFX (28.1%), and VDZ (25.3%). The overall switch rate over 3 years of follow-up was highest for IFX (32.9%), followed by ADA (26.6%), VDZ (24.5%), and UST (18.8%). Switches within the allowable gap showed the same trend as for the overall switch rate; the proportion of patients varied in range from 5.1% (for UST) to 18.5% (for IFX). For switches after the allowable gap, 18.9% of ADA patients switched treatment, followed by IFX (14.4%), UST (13.7%), and VDZ (11.9%). Patients who discontinued their index therapy but restarted it after the non-allowable gap were highest among IFX and ADA (both 24.3%), followed by UST (22.6%) and VDZ (21.6%).

**Adherence**: The proportion of patients who had an MPR of at least 80% based on treatment duration were highest for ADA (90.6%) followed by UST (86.5%), VDZ (85.0%), and IFX (79.5%). The average PDC based on treatment duration was highest for ADA (0.92 ± 0.10; median, 0.95) followed by UST (0.91 ± 0.16; median, 1.00), VDZ (0.90 ± 0.15; median, 0.95), and IFX (0.87 ± 0.16; median, 0.93) (**Table 2**).

**Table 2.** Treatment Patterns Among Crohn’s Disease Patients Using Biologics With 3 Years of Follow-Up

	**Biologics**							
	**UST (n = 394)**		**VDZ (n = 486)**		**IFX (n = 847)**		**ADA (n = 534)**	
	**N/Mean**	**%/SD**	**N/Mean**	**%/SD**	**N/Mean**	**%/SD**	**N/Mean**	**%/SD**
Sample size	**394**		486		847		534	
Persistence	242	61.4%	282	58.0%	407	48.1%	278	52.1%
Overall discontinuation	152	38.6%	204	42.0%	440	52.0%	256	47.9%
Overall switch	74	18.8%	119	24.5%	279	32.9%	142	26.6%
Switch within allowable gap	20	5.1%	61	12.6%	157	18.5%	41	7.7%
ADA	2	0.5%	13	2.7%	5	0.6%	0	0.0%
CZP	3	0.8%	1	0.2%	0	0.0%	5	0.9%
IFX	6	1.5%	6	1.2%	0	0.0%	2	0.4%
IFX BS	1	0.3%	2	0.4%	77	9.1%	0	0.0%
UST	0	0.0%	38	7.8%	29	3.4%	23	4.3%
VDZ	8	2.0%	1	0.2%	45	5.3%	11	2.1%
Switch after allowable gap	54	13.7%	58	11.9%	122	14.4%	101	18.9%
ADA	0	0.0%	0	0.0%	9	1.1%	0	0.0%
CZP	3	0.8%	2	0.4%	2	0.2%	4	0.8%
IFX	10	2.5%	21	4.3%	0	0.0%	25	4.7%
IFX-BS	2	0.5%	1	0.2%	38	4.5%	2	0.4%
UST	0	0.0%	28	5.8%	35	4.1%	35	6.6%
VDZ	34	8.6%	0	0.0%	38	4.5%	32	6.0%
Restart of index biologic	89	22.6%	105	21.6%	206	24.3%	130	24.3%
Discontinuation without restart or switch	121	30.7%	123	25.3%	238	28.1%	199	37.3%
Medication adherence (MPR) based on treatment duration								
MPR ≥80%	341	86.6%	413	85.0%	673	79.5%	484	90.6%
Medication adherence (MPR) based on length of follow-up								
MPR ≥ 80%	35	8.9%	94	19.3%	115	13.6%	40	7.5%
PDC based on treatment duration								
Mean	0.9	0.2	0.9	0.2	0.9	0.2	0.9	0.1
Median (IQR)	1.0 (0.88-1.0)		1.0 (0.86-1.0)		0.9 (0.81-1.0)		1.0 (0.87-1.0)	
PDC based on length of follow-up								
Mean	0.2	0.3	0.3	0.3	0.3	0.3	0.2	0.3
Median (IQR)	0.1 (0.04-0.27)		0.2 (0.07-0.32)		0.2 (0.05-0.46)		0.1 (0.03-0.33)	

Abbreviations: ADA, adalimumab (Humira), CZP, certolizumab pegol (Cimzia), IFX, infliximab (Remicade); IQR, interquartile range; MPR, medication possession ratio; NTZ, natalizumab; PDC, proportion of days covered; UST, ustekinumab (Stelara); VDZ, edolizumab (Entyvio).

In contrast, ADA had the lowest proportion of patients with an MPR of at least 80% based on follow-up period (7.49%), followed by 8.9% for UST, 13.6% for IFX, and 19.3% for VDZ, which was the highest. The average PDC based on follow-up time was highest for VDZ (0.33 ± 0.32; median, 0.19) followed by IFX (0.30 ± 0.30; median, 0.19), ADA (0.22 ± 0.25; median, 0.10), and UST (0.21 ± 0.27; median, 0.05) (**Table 2**).

**Dose titration:** Among patients with 3 years of follow-up, the number of eligible patients (those who had maintenance therapy with at least 2 maintenance doses identified by the same type of claims during the follow-up period) were 367 (68.7%) for ADA, 648 (76.5%) for IFX, 200 (50.8%) for UST, and 362 (74.5%) for VDZ (**Table 3**).

**Table 3.** Dose Titration Among CD Patients Using Biologics Over 3 Years of Follow-Up

**Study Period: September 26, 2015–October 31, 2021**		
**Identification Period: September 26, 2016–October 31, 2018**		
**Biologics**	**N**	**Percentage (%)**
ADA sample size	**534**	
Eligible cases	**367**	**68.7**
Dose reduction	19	5.2
0-50%	14	3.8
>50%	5	1.4
Dose escalation	174	47.4
≤50%	11	3.0
51%-100%	118	32.2
>100%	45	12.3
Unchanged	174	47.4
IFX sample size	**847**	
Eligible cases	**648**	**76.5**
Dose reduction	74	11.4
≤50%	43	6.6
>50%	31	4.8
Dose escalation	427	65.9
≤50%	54	8.3
51%-100%	107	16.5
>100%	266	41.1
Unchanged	147	22.7
UST sample size	**394**	
Eligible cases	**200**	**50.8**
Dose reduction	33	16.5
≤50%	32	16.0
>50%	1	0.5
Dose escalation	71	35.5
≤50%	26	13.0
51%-100%	28	14.0
>100%	17	8.5
Unchanged	96	48.0
VDZ sample size	**486**	
Eligible cases	**362**	**74.5**
Dose reduction	53	14.6
≤50%	32	8.8
>50%	21	5.8
Dose escalation	193	53.3
≤50%	14	3.9
51%-100%	84	23.2
>100%	95	26.2
Unchanged	116	32.0

Two types of percentages were reported: (1) eligible patients’ percentage are reported with respect to total sample size of each biologic as the denominator and (2) dose titration–related patients’ percentage are reported with respect to eligible patients for each biologic as the denominator.

Abbreviations: ADA, adalimumab; CZP, certolizumab pegol; IFX, infliximab; UST, ustekinumab; VDZ, vedolizumab.

Among eligible patients for each biologic, the proportion of patients undergoing any dose titration was highest for IFX (77.3%), followed by VDZ (68.0%), ADA (52.6%), and UST (52.0%). For patients with dose titration, UST was observed with the highest proportion of dose-reduction (16.5%) and IFX was observed with the highest proportion of dose escalation (65.9%). In particular, patients treated with IFX had the highest proportion of a >100% of dose escalation (41.1%) while patients treated with UST had the lowest proportion (8.5%). Detailed dose titration results across biologics are shown in **Table 3**.

## DISCUSSION

Our results showed that more patients received IFX, ADA, and VDZ than UST, likely reflecting their position as more established treatments. UST is the most recent biologic approved by the FDA (September 23, 2016), so it is not surprising to see limited use among patients with CD. Although it has been on the market for the shortest time, according to our study, patients on UST had the highest persistence rate and lowest dose escalation rate across different biologic users over the 3 years of follow-up, possibly suggesting a better clinical response of UST.

To our knowledge, this is the first study to descriptively assess the treatment patterns of commonly prescribed biologics for CD over a 3-year follow-up period using an all-payer administrative claims database representing 80% of the US healthcare system. Leveraging RWD Insights database capture of individuals longitudinally over different payer channels and health plans, this retrospective observational study provides a current summary of the real-world use of biologics for CD in the United States by exploring biologic persistence, adherence, and dose titration.

Our results suggest that biologics differed to the degree in which patients persisted on their treatment regimen. Patients on UST and VDZ showed numerically higher persistence than ADA and IFX. Persistence was above 50% (52.1%-61.4%) for all biologics except for IFX, which was 48.1%. These findings are comparable to previous studies showing that 28% to 62% of patients were persistent on anti-TNF biologics.^18,25,26^ Other studies have reported higher persistence rates of biologics.^21,22,27^ For example, a recent CD study using pooled US commercial claims data sources (IBM MarketScan®, IQVIA PharMetrics®, and Optum) reported a similar trend with highest persistence among both biologic-naïve and biologic-experienced patients with CD for UST (87.2%; 86.3%), followed by VDZ (78.9%; 80.8%), IFX (79.0%; 77.4%), and ADA (64.9%; 60.7%).^21^ Another recent study with real-world data from 12 hospitals in Finland reported a 83.3% persistence rate for UST during a 12-month follow-up period. The higher persistence rates found in these studies may be attributed to the shorter follow-up period (1 year) compared with our study, with 3 years of follow-up. A decrease in persistence to UST over time was reported in a recently published real-world evidence study on adult patients CD in Israel. Using the second-largest state-mandated health care provider database in Israel, researchers observed lower persistence to UST over time with 89.4%, 72.2%, and 64.4% at 180, 365, and 540 days after UST induction, respectively.

In particular, the current study showed UST patients had the lowest discontinuation rate (38.6%) and were less likely to switch to other biologics (18.8%) than patients who used other biologics. Our results are consistent with other research findings.^21,22,28^ Discontinuation/switch rates for biologics can serve as useful proxies for their effectiveness in patients with CD.^15,25,29^ All clinical trials with UST have reported significantly improved clinical responses than placebo among patients with CD who previously failed or were intolerant to anti-TNF biologics.^30^ The lowest discontinuation and switch rates of UST observed across different biologics in the current study provides real-world evidence on UST’s effectiveness. As one of the most recent FDA-approved biologics for CD, real-world evidence on UST is limited. Further real-world evidence studies comparing UST to other biologics are needed to assess persistence rates over more extended follow-up periods.

Another useful proxy for biologic effectiveness among patients with CD is dose titration. Dose titration is done to change the mediation to achieve the best clinical response. This can occur by dose escalation with increasing the dose of a medication over time for effective or by dose reduction with decreasing the dose of a medication over time to lessen adverse effects. Dose titration is specifically pertinent to the biologics’ usage over a longer follow-up period as these biologics tend to impact the humanistic and economic outcomes. 16-20 In the current study, the dose titration rate was highest for IFX (76.5%), followed by VDZ (74.5%), and ADA (68.7%), and lowest for UST (50.8%) over the 3 years of follow-up. Overall, more patients had dose escalation than dose reduction, indicating more patients tolerated their starting dose but needed a higher dose for effectiveness.

Patients with UST use had the lowest overall dose escalation rate, mainly driven by its low rate in >100% does escalation. Approximately 1 of 3 patients who initiated UST had a dose increase over the 3-year follow up, compared with 1 of 2 patients using other biologics. Specifically, UST has the lowest proportion of patients with a >100% increase of the initial dosage (8.3%), while IFX had the highest proportion (41.4%). These results align with the observation that patients on UST had the highest persistence, possibly indicating better effectiveness. The highest dose reduction for UST observed in our study further acknowledge other study’s finding.^21^ This could be attributed to the selection bias of UST patients intolerant to biologics. It is well-known that UST has been recommended to treat patients with CD who failed or were intolerant to anti-TNF biologics. Teeple et al reported that a higher proportion of UST patients were treated with 2 or more biologics prior to UST initiation. In addition, it is possible that patients with UST use were refractory patients with more severe disease, which would be acknowledged by the highest CCI score for UST patients in our study. Thus, intolerance to biologics and a more severe disease status can lead to high dose reduction rates in UST patients. Future studies would be needed to investigate the tolerability to UST and related adverse effects.

The approval of new biologics continues to change the treatment landscape for patients with CD. This study adds to existing literature by describing the treatment patterns of commonly prescribed biologics in a real-world cohort of patients with CD with an extended follow-up period. In particular, this study provides a much needed and updated summary of biologic therapy in US clinical practice with inclusion of the most recently approved biologic, which is not well-captured in the existing literature. It used a nationwide claim database (RWD Insights) that tracked individuals longitudinally and provided a more comprehensive coverage of patient populations across all US commercial insurances and provider groups. Because of variability of patient and provider characteristics in the database, generalizability of the study results to the public is improved. Also, as patient consent is not necessary for collecting administrative claims data, related biases are alleviated.

Nevertheless, the study findings should be interpreted with consideration of some limitations inherent to claims data. First, as with all retrospective observational analyses, this study was limited to determining associations, and causality cannot be inferred. As this study was descriptive and no tests of significance were conducted in this study, results should be interpreted with caution when comparing biologics. Second, claims data may be subject to medical coding errors or inaccuracies. For example, the presence of a claim for a filled prescription does not indicate whether the medication was consumed or taken as prescribed. Medication claims filled over the counter or provided as samples by the physician are not observed in the claims data. This may result in an overestimate or underestimate of persistence. Furthermore, certain information that could influence study outcomes, such as clinical and disease-specific information, is not readily available in claims data. For example, the reasons for drug discontinuation or switch are not documented in claims database. Thus, factors influencing treatment patterns were not clear. Finally, since our study population was commercially insured, our results might not be generalizable to US non-commercially insured patients and patients outside the United States. Few of these drugs were being introduced around the time of our research, thereby becoming accessible through commercial insurance. Furthermore, to minimize the impact of potential confounding factors and variations stemming from challenges associated with different insurance types, our emphasis has been on patients covered by commercial insurance. Future real-world studies adjusting for potential confounders and comparing the treatment effectiveness and adverse event rates across different biologics should be conducted to assist stakeholders in making informed decisions regarding treatment strategies for patients with CD.

Sample size for certain groups was a major limitation for this study. Due to the small sample size for the biologic-experienced group, results across biologic-naïve and biologic-experienced patients could not be stratified. Stratification is important to understand the inherent variability between patients in each group. Compared with the biologic-naïve group, biologic-experienced patients had an inherent bias introduced from prior biologic treatment exposure and potential nonresponse or failure that may have led to initiation of a new biologic therapy. Future studies that may involve larger data pools of biologic-experienced patients are warranted.

## CONCLUSION

This large, retrospective, real-world study using nationwide claims data for commercially-insured patients with CD described persistence, discontinuation, and dose titration of commonly prescribed biologics. Over the 3 years of follow-up, persistence was highest in the UST cohort. In addition, the UST cohort had the lowest proportion of patients with dose titration. In particular, the UST cohort had the highest proportion of patients with dose-reduction and the lowest proportion of patients with dose-escalation, possibly indicating better clinical responses of UST. Our study further acknowledges the fact that longer periods of observation should be considered for future studies with adjustment for confounders to better understand treatment pattern across biologics over time.

### Author Contributions

All authors participated in the conception and design of the study, the analysis and interpretation of the data, the drafting of the paper or revising it critically for intellectual content, and for approval of the definitive version to be published. All authors agree to be accountable for all aspects of the work.

### Declarations

R.Z., Z.D., and S.K. are employees of Janssen Scientific Affairs, LLC, and Johnson & Johnson. P.G., L.G., R.B., V.M.J., Y.L., and K.M. are employees of STATinMED, LLC and supported this study as a paid consultant to Janssen Scientific Affairs.

### Data Availability

Due to the data user agreement between the authors and the data vendor, data cannot be made publicly available.

## Supplementary Material

Online Supplementary Material
