# Supplementary material for: Evaluation of Treatment Patterns and Maintenance Dose Titration Among Patients With Crohn’s Disease Initiating Biologics With 3 Years of Follow-Up
Source: J Health Econ Outcomes Res. 2023 Nov 20;10(2):111–20. doi: 10.36469/001c.88947 (PMC10664831; doi:10.36469/001c.88947)
Supplement: Online Supplementary Material [file jheor_2023_10_2_88947_186275.pdf]

APPENDIX: MARKET DEFINITIONS

Crohn’s Disease

DIAG_FMT	CODE_TYPE	DIAG_DESC
5550	ICD9	Regional enteritis of small intestine
5551	ICD9	Regional enteritis of large intestine
5552	ICD9	Regional enteritis of small intestine with large intestine
5559	ICD9	Regional enteritis of unspecified site
K500	ICD10	Crohn's disease of small intestine
K5000	ICD10	Crohn's disease of small intestine without complications
K50011	ICD10	Crohn's disease of small intestine with rectal bleeding
K50012	ICD10	Crohn's disease of small intestine with intestinal obstruction
K50013	ICD10	Crohn's disease of small intestine with fistula
K50014	ICD10	Crohn's disease of small intestine with abscess
K50018	ICD10	Crohn's disease of small intestine with other complication
K50019	ICD10	Crohn's disease of small intestine with unspecified complications
K501	ICD10	Crohn's disease of large intestine
K5010	ICD10	Crohn's disease of large intestine without complications
K50111	ICD10	Crohn's disease of large intestine with rectal bleeding
K50112	ICD10	Crohn's disease of large intestine with intestinal obstruction
K50113	ICD10	Crohn's disease of large intestine with fistula
K50114	ICD10	Crohn's disease of large intestine with abscess
K50118	ICD10	Crohn's disease of large intestine with other complication
K50119	ICD10	Crohn's disease of large intestine with unspecified complications
K508	ICD10	Crohn's disease of both small and large intestine
K5080	ICD10	Crohn's disease of both small and large intestine without complication
K50811	ICD10	Crohn's disease of both small and large intestine with rectal bleeding
K50812	ICD10	Crohn's disease of both small and large intestine with intestinal obstruction
K50813	ICD10	Crohn's disease of both small and large intestine with fistula
K50814	ICD10	Crohn's disease of both small and large intestine with abscess
K50818	ICD10	Crohn's disease of both small and large intestine with other complication
K50819	ICD10	Crohn's disease of both small and large intestine with unspecified com
K509	ICD10	Crohn's disease, unspecified
K5090	ICD10	Crohn's disease unspecified without complications
K50911	ICD10	Crohn's disease unspecified with rectal bleeding
K50912	ICD10	Crohn's disease unspecified with intestinal obstruction
K50913	ICD10	Crohn's disease unspecified with fistula
K50914	ICD10	Crohn's disease unspecified with abscess
K50918	ICD10	Crohn's disease unspecified with other complication
K50919	ICD10	Crohn's disease unspecified with unspecified complications

Remicade

RX_FMT	RX_CODE_TYPE	RX_DESC
J1745	HCPCS	Injection, infliximab, excludes biosimilar, 10 mg
57894003001	NDC	Infliximab For IV Inj 100 MG - REMICADE Intravenous Injection, Powder, Lyophilized, For Solution - 100 Mg/10ml Infliximab
57894016001	NDC	Infliximab For IV Inj 100 MG - INFLIXIMAB Intravenous Injection, Powder, Lyophilized, For Solution - 100 Mg/10ml Infliximab
S9359	HCPCS	Home infusion therapy, anti-tumor necrosis factor intravenous therapy; (e.g., Infliximab); administrative services, professional pharmacy services, care coordination, and all necessary supplies and equipment (drugs and nursing visits coded separately)

Remicade Biosimilar

RX_FMT	RX_CODE_TYPE	RX_DESC
Q5109	HCPCS	Injection, infliximab-qbtx, biosimilar, (ixifi), 10 mg
Q5102	HCPCS	Injection, infliximab, biosimilar, 10 mg
Q5103	HCPCS	Injection, infliximab-dyyb, biosimilar, (inflectra), 10 mg
Q5104	HCPCS	Injection, infliximab-abda, biosimilar, (renflexis), 10 mg
Q5121	HCPCS	Injection, infliximab-axxq, biosimilar, (avsola), 10 mg
00069080901	NDC	Infliximab-dyyb For IV Inj 100 MG - INFLECTRA Intravenous Injection, Powder, Lyophilized, For Solution - 100 Mg/10ml Infliximab
00006430502	NDC	Infliximab-abda For IV Inj 100 MG - Renflexis Intravenous Injection, Powder, Lyophilized, For Solution - 100 Mg/1 Infliximab
00006430501	NDC	RENFLEXIS
00069080901	NDC	INFLECTRA
55513067001	NDC	AVSOLA
78206-016201	NDC	RENFLEXIS
78206016299	NDC	RENFLEXIS

Natalizumab

RX_FMT	RX_CODE_TYPE	RX_DESC
J2323	HCPCS	Natalizumab
Q4079	HCPCS	Natalizumab
C9126	HCPCS	Natalizumab
59075073015	NDC	TYSABRI
64406000801	NDC	TYSABRI

Other Biologics

RX_FMT	RX_CODE_TYPE	RX_DESC
J0135	HCPCS	Adalimumab
J0718	HCPCS	Certolizumab
J0717	HCPCS	Certolizumab
C9249	HCPCS	Certolizumab

RX_FMT	RX_CODE_TYPE	RX_DESC
C9026	HCPCS	Vedolizumab
J3380	HCPCS	Vedolizumab
C9261	HCPCS	Ustekinumab
J3357	HCPCS	Ustekinumab
C9487	HCPCS	Ustekinumab
J3358	HCPCS	Ustekinumab
Q9989	HCPCS	Ustekinumab
00074006702	NDC	Adalimumab
00074012401	NDC	Adalimumab
00074012402	NDC	Adalimumab
00074012403	NDC	Adalimumab
00074012404	NDC	Adalimumab
00074012473	NDC	Adalimumab
00074012474	NDC	Adalimumab
00074024302	NDC	Adalimumab
00074024371	NDC	Adalimumab
00074055401	NDC	Adalimumab
00074055402	NDC	Adalimumab
00074055404	NDC	Adalimumab
00074055406	NDC	Adalimumab
00074055471	NDC	Adalimumab
00074055473	NDC	Adalimumab
00074055474	NDC	Adalimumab
00074061602	NDC	Adalimumab
00074061671	NDC	Adalimumab
00074081702	NDC	Adalimumab
00074153903	NDC	Adalimumab
00074254001	NDC	Adalimumab
00074254003	NDC	Adalimumab
00074379901	NDC	Adalimumab
00074379902	NDC	Adalimumab
00074379903	NDC	Adalimumab
00074379906	NDC	Adalimumab
00074379971	NDC	Adalimumab
00074433901	NDC	Adalimumab
00074433902	NDC	Adalimumab
00074433906	NDC	Adalimumab
00074433907	NDC	Adalimumab
00074433971	NDC	Adalimumab
00074433973	NDC	Adalimumab
00074433974	NDC	Adalimumab
00074634702	NDC	Adalimumab
00074937402	NDC	Adalimumab
00074937471	NDC	Adalimumab

RX_FMT	RX_CODE_TYPE	RX_DESC
50090353000	NDC	Adalimumab
54569552400	NDC	Adalimumab
54868482200	NDC	Adalimumab
50474070061	NDC	Certolizumab
50474070062	NDC	Certolizumab
50474071079	NDC	Certolizumab
50474071080	NDC	Certolizumab
50474071081	NDC	Certolizumab
57894005416	NDC	Ustekinumab
57894005427	NDC	Ustekinumab
57894006002	NDC	Ustekinumab
57894006003	NDC	Ustekinumab
57894006004	NDC	Ustekinumab
57894006103	NDC	Ustekinumab
57894006104	NDC	Ustekinumab
99999991019	NDC	Ustekinumab
64764030020	NDC	Vedolizumab
00074006702	NDC	Adalimumab

Exclusion Criteria

DIAG_FMT	CODE_TYPE	DIAG_DESC
446	ICD9	Polyarteritis nodosa and allied conditions
556	ICD9	Ulcerative enterocolitis
696	ICD9	Psoriasis and similar disorders
714	ICD9	Rheumatoid arthritis and other inflammatory polyarthropathies
720	ICD9	Ankylosing spondylitis and other inflammatory spondylopathies
725	ICD9	Polymyalgia rheumatica
4460	ICD9	Polyarteritis nodosa
4461	ICD9	Acute febrile mucocutaneous lymph node syndrome
4462	ICD9	Hypersensitivity angiitis
4463	ICD9	Lethal midline granuloma
4464	ICD9	Wegener's granulomatosis
4465	ICD9	Giant cell arteritis
4466	ICD9	Thrombotic microangiopathy
4467	ICD9	Takayasu's disease
5560	ICD9	Ulcerative (chronic) enterocolitis
5561	ICD9	Ulcerative (chronic) ileocolitis
5562	ICD9	Ulcerative (chronic) proctitis
5563	ICD9	Ulcerative (chronic) proctosigmoiditis
5564	ICD9	Pseudopolyposis of colon
5565	ICD9	Left-side ulcerative (chronic) colitis
5566	ICD9	Universal ulcerative (chronic) colitis
5568	ICD9	Other ulcerative colitis

DIAG_FMT	CODE_TYPE	DIAG_DESC
5569	ICD9	Ulcerative colitis, unspecified
6960	ICD9	Psoriatic arthropathy
6961	ICD9	Other psoriasis
6962	ICD9	Parapsoriasis
6963	ICD9	Pityriasis rosea
6964	ICD9	Pityriasis rubra pilaris
6965	ICD9	Other and unspecified pityriasis
6968	ICD9	Other psoriasis and similar disorders
7010	ICD9	Circumscribed scleroderma
7100	ICD9	Systemic lupus erythematosus
7101	ICD9	Systemic sclerosis
7102	ICD9	Sicca syndrome
7103	ICD9	Dermatomyositis
7104	ICD9	Polymyositis
7108	ICD9	Other specified diffuse diseases of connective tissue
7109	ICD9	Unspecified diffuse connective tissue disease
7112	ICD9	Arthropathy in behcet's syndrome
7140	ICD9	Rheumatoid arthritis
7141	ICD9	Felty's syndrome
7142	ICD9	Other rheumatoid arthritis with visceral or systemic involvement
7143	ICD9	Juvenile chronic polyarthritis
7144	ICD9	Chronic postrheumatic arthropathy
7148	ICD9	Other specified inflammatory polyarthropathies
7149	ICD9	Unspecified inflammatory polyarthropathy
7193	ICD9	Palindromic rheumatism
7200	ICD9	Ankylosing spondylitis
7201	ICD9	Spinal enthesopathy
7202	ICD9	Sacroiliitis, not elsewhere classified
7208	ICD9	Other inflammatory spondylopathies
7209	ICD9	Unspecified inflammatory spondylopathy
7285	ICD9	Hypermobility syndrome
44620	ICD9	Hypersensitivity angiitis, unspecified
44621	ICD9	Goodpasture's syndrome
44629	ICD9	Other specified hypersensitivity angiitis
71120	ICD9	Arthropathy in behcet's syndrome, site unspecified
71121	ICD9	Arthropathy in behcet's syndrome, shoulder region
71122	ICD9	Arthropathy in behcet's syndrome, upper arm
71123	ICD9	Arthropathy in behcet's syndrome, forearm
71124	ICD9	Arthropathy in behcet's syndrome, hand
71125	ICD9	Arthropathy in behcet's syndrome, pelvic region, and thigh
71126	ICD9	Arthropathy in behcet's syndrome, lower leg
71127	ICD9	Arthropathy in behcet's syndrome, ankle, and foot
71128	ICD9	Arthropathy in behcet's syndrome, other specified sites
71129	ICD9	Arthropathy in behcet's syndrome, multiple sites

DIAG_FMT	CODE_TYPE	DIAG_DESC
71430	ICD9	Polyarticular juvenile rheumatoid arthritis, chronic or unspecified
71431	ICD9	Polyarticular juvenile rheumatoid arthritis, acute
71432	ICD9	Pauciarticular juvenile rheumatoid arthritis
71433	ICD9	Monoarticular juvenile rheumatoid arthritis
71481	ICD9	Rheumatoid lung
71489	ICD9	Other specified inflammatory polyarthropathies
71930	ICD9	Palindromic rheumatism, site unspecified
71931	ICD9	Palindromic rheumatism, shoulder region
71932	ICD9	Palindromic rheumatism, upper arm
71933	ICD9	Palindromic rheumatism, forearm
71934	ICD9	Palindromic rheumatism, hand
71935	ICD9	Palindromic rheumatism, pelvic region, and thigh
71936	ICD9	Palindromic rheumatism, lower leg
71937	ICD9	Palindromic rheumatism, ankle, and foot
71938	ICD9	Palindromic rheumatism, other specified sites
71939	ICD9	Palindromic rheumatism, multiple sites
72081	ICD9	Inflammatory spondylopathies in diseases classified elsewhere
72089	ICD9	Other inflammatory spondylopathies
72889	ICD9	Other disorders of muscle, ligament, and fascia
72930	ICD9	Panniculitis, unspecified site
630	ICD9	Ectopic And Molar Pregnancy and Other Pregnancy with Abortive Outcome
631	ICD9	Ectopic And Molar Pregnancy and Other Pregnancy with Abortive Outcome
632	ICD9	Ectopic And Molar Pregnancy and Other Pregnancy with Abortive Outcome
633	ICD9	Ectopic And Molar Pregnancy and Other Pregnancy with Abortive Outcome
634	ICD9	Ectopic And Molar Pregnancy and Other Pregnancy with Abortive Outcome
635	ICD9	Ectopic And Molar Pregnancy and Other Pregnancy with Abortive Outcome
636	ICD9	Ectopic And Molar Pregnancy and Other Pregnancy with Abortive Outcome
637	ICD9	Ectopic And Molar Pregnancy and Other Pregnancy with Abortive Outcome
638	ICD9	Ectopic And Molar Pregnancy and Other Pregnancy with Abortive Outcome
639	ICD9	Ectopic And Molar Pregnancy and Other Pregnancy with Abortive Outcome
640	ICD9	Complications Mainly Related to Pregnancy
641	ICD9	Complications Mainly Related to Pregnancy
642	ICD9	Complications Mainly Related to Pregnancy
643	ICD9	Complications Mainly Related to Pregnancy
644	ICD9	Complications Mainly Related to Pregnancy
645	ICD9	Complications Mainly Related to Pregnancy
646	ICD9	Complications Mainly Related to Pregnancy
647	ICD9	Complications Mainly Related to Pregnancy
648	ICD9	Complications Mainly Related to Pregnancy
649	ICD9	Complications Mainly Related to Pregnancy
650	ICD9	Normal Delivery, And Other Indications for Care in Pregnancy, Labor, And Delivery
651	ICD9	Normal Delivery, And Other Indications for Care in Pregnancy, Labor, And Delivery
652	ICD9	Normal Delivery, And Other Indications for Care in Pregnancy, Labor, And Delivery

DIAG_FMT	CODE_TYPE	DIAG_DESC
653	ICD9	Normal Delivery, And Other Indications for Care in Pregnancy, Labor, And Delivery
654	ICD9	Normal Delivery, And Other Indications for Care in Pregnancy, Labor, And Delivery
655	ICD9	Normal Delivery, And Other Indications for Care in Pregnancy, Labor, And Delivery
656	ICD9	Normal Delivery, And Other Indications for Care in Pregnancy, Labor, And Delivery
657	ICD9	Normal Delivery, And Other Indications for Care in Pregnancy, Labor, And Delivery
658	ICD9	Normal Delivery, And Other Indications for Care in Pregnancy, Labor, And Delivery
659	ICD9	Normal Delivery, And Other Indications for Care in Pregnancy, Labor, And Delivery
660	ICD9	Complications Occurring Mainly in The Course of Labor and Delivery
661	ICD9	Complications Occurring Mainly in The Course of Labor and Delivery
662	ICD9	Complications Occurring Mainly in The Course of Labor and Delivery
663	ICD9	Complications Occurring Mainly in The Course of Labor and Delivery
664	ICD9	Complications Occurring Mainly in The Course of Labor and Delivery
665	ICD9	Complications Occurring Mainly in The Course of Labor and Delivery
666	ICD9	Complications Occurring Mainly in The Course of Labor and Delivery
667	ICD9	Complications Occurring Mainly in The Course of Labor and Delivery
668	ICD9	Complications Occurring Mainly in The Course of Labor and Delivery
669	ICD9	Complications Occurring Mainly in The Course of Labor and Delivery
670	ICD9	Complications Of the Puerperium
671	ICD9	Complications Of the Puerperium
672	ICD9	Complications Of the Puerperium
673	ICD9	Complications Of the Puerperium
674	ICD9	Complications Of the Puerperium
675	ICD9	Complications Of the Puerperium
676	ICD9	Complications Of the Puerperium
677	ICD9	Complications Of the Puerperium
678	ICD9	Other Maternal and Fetal Complications
679	ICD9	Other Maternal and Fetal Complications
73399	ICD9	Other disorders of bone and cartilage
K5180	ICD10	Other ulcerative colitis without complications
K51811	ICD10	Other ulcerative colitis with rectal bleeding
K51812	ICD10	Other ulcerative colitis with intestinal obstruction
K51813	ICD10	Other ulcerative colitis with fistula
K51814	ICD10	Other ulcerative colitis with abscess
K51818	ICD10	Other ulcerative colitis with other complication
K51819	ICD10	Other ulcerative colitis with unspecified complications
K5190	ICD10	Ulcerative colitis, unspecified, without complications
K51911	ICD10	Ulcerative colitis, unspecified with rectal bleeding
K51912	ICD10	Ulcerative colitis, unspecified with intestinal obstruction
K51913	ICD10	Ulcerative colitis, unspecified with fistula
K51914	ICD10	Ulcerative colitis, unspecified with abscess
K51918	ICD10	Ulcerative colitis, unspecified with other complication

DIAG_FMT	CODE_TYPE	DIAG_DESC
K51919	ICD10	Ulcerative colitis, unspecified with unspecified complications
L400	ICD10	Psoriasis vulgaris
L401	ICD10	Generalized pustular psoriasis
L404	ICD10	Guttate psoriasis
L4050	ICD10	Arthropathic psoriasis, unspecified
L408	ICD10	Other psoriasis
L409	ICD10	Psoriasis, unspecified
L413	ICD10	Small plaque parapsoriasis
L414	ICD10	Large plaque parapsoriasis
L415	ICD10	Retiform parapsoriasis
L418	ICD10	Parapsoriasis
L419	ICD10	Parapsoriasis, unspecified
L940	ICD10	Localized scleroderma [morphea]
L941	ICD10	Linear scleroderma
L943	ICD10	Sclerodactyly
M05	ICD10	Rheumatoid arthritis with rheumatoid factor
M050	ICD10	Felty's syndrome
M0500	ICD10	Felty's syndrome, unspecified site
M0501	ICD10	Felty's syndrome, shoulder
M05011	ICD10	Felty's syndrome, right shoulder
M05012	ICD10	Felty's syndrome, left shoulder
M05019	ICD10	Felty's syndrome, unspecified shoulder
M0502	ICD10	Felty's syndrome, elbow
M05021	ICD10	Felty's syndrome, right elbow
M05022	ICD10	Felty's syndrome, left elbow
M05029	ICD10	Felty's syndrome, unspecified elbow
M0503	ICD10	Felty's syndrome, wrist
M05031	ICD10	Felty's syndrome, right wrist
M05032	ICD10	Felty's syndrome, left wrist
M05039	ICD10	Felty's syndrome, unspecified wrist
M0504	ICD10	Felty's syndrome, hand
M05041	ICD10	Felty's syndrome, right hand
M05042	ICD10	Felty's syndrome, left hand
M05049	ICD10	Felty's syndrome, unspecified hand
M0505	ICD10	Felty's syndrome, hip
M05051	ICD10	Felty's syndrome, right hip
M05052	ICD10	Felty's syndrome, left hip
M05059	ICD10	Felty's syndrome, unspecified hip
M0506	ICD10	Felty's syndrome, knee
M05061	ICD10	Felty's syndrome, right knee
M05062	ICD10	Felty's syndrome, left knee
M05069	ICD10	Felty's syndrome, unspecified knee
M0507	ICD10	Felty's syndrome, ankle, and foot
M05071	ICD10	Felty's syndrome, right ankle, and foot
M05072	ICD10	Felty's syndrome, left ankle and foot
M05079	ICD10	Felty's syndrome, unspecified ankle, and foot

DIAG_FMT	CODE_TYPE	DIAG_DESC
M0509	ICD10	Felty's syndrome, multiple sites
M051	ICD10	Rheumatoid lung disease with rheumatoid arthritis
M0510	ICD10	Rheumatoid lung disease w rheumatoid arthritis of unsp site
M0511	ICD10	Rheumatoid lung disease w rheumatoid arthritis of shoulder
M05111	ICD10	Rheumatoid lung disease w rheumatoid arthritis of r shoulder
M05112	ICD10	Rheumatoid lung disease w rheumatoid arthritis of l shoulder
M05119	ICD10	Rheu lung disease w rheumatoid arthritis of unsp shoulder
M0512	ICD10	Rheumatoid lung disease with rheumatoid arthritis of elbow
M05121	ICD10	Rheumatoid lung disease w rheumatoid arthritis of r elbow
M05122	ICD10	Rheumatoid lung disease w rheumatoid arthritis of left elbow
M05129	ICD10	Rheumatoid lung disease w rheumatoid arthritis of unsp elbow
M0513	ICD10	Rheumatoid lung disease with rheumatoid arthritis of wrist
M05131	ICD10	Rheumatoid lung disease w rheumatoid arthritis of r wrist
M05132	ICD10	Rheumatoid lung disease w rheumatoid arthritis of left wrist
M05139	ICD10	Rheumatoid lung disease w rheumatoid arthritis of unsp wrist
M0514	ICD10	Rheumatoid lung disease with rheumatoid arthritis of hand
M05141	ICD10	Rheumatoid lung disease w rheumatoid arthritis of right hand
M05142	ICD10	Rheumatoid lung disease w rheumatoid arthritis of left hand
M05149	ICD10	Rheumatoid lung disease w rheumatoid arthritis of unsp hand
M0515	ICD10	Rheumatoid lung disease with rheumatoid arthritis of hip
M05151	ICD10	Rheumatoid lung disease w rheumatoid arthritis of right hip
M05152	ICD10	Rheumatoid lung disease w rheumatoid arthritis of left hip
M05159	ICD10	Rheumatoid lung disease w rheumatoid arthritis of unsp hip
M0516	ICD10	Rheumatoid lung disease with rheumatoid arthritis of knee
M05161	ICD10	Rheumatoid lung disease w rheumatoid arthritis of right knee
M05162	ICD10	Rheumatoid lung disease w rheumatoid arthritis of left knee
M05169	ICD10	Rheumatoid lung disease w rheumatoid arthritis of unsp knee
M0517	ICD10	Rheumatoid lung disease w rheumatoid arthritis of ank/ft
M05171	ICD10	Rheu lung disease w rheumatoid arthritis of right ank/ft
M05172	ICD10	Rheu lung disease w rheumatoid arthritis of left ank/ft
M05179	ICD10	Rheu lung disease w rheumatoid arthritis of unsp ank/ft
M0519	ICD10	Rheumatoid lung disease w rheumatoid arthritis mult site
M052	ICD10	Rheumatoid vasculitis with rheumatoid arthritis
M0520	ICD10	Rheumatoid vasculitis with rheumatoid arthritis of unsp site
M0521	ICD10	Rheumatoid vasculitis with rheumatoid arthritis of shoulder
M05211	ICD10	Rheumatoid vasculitis w rheumatoid arthritis of r shoulder
M05212	ICD10	Rheumatoid vasculitis w rheumatoid arthritis of l shoulder
M05219	ICD10	Rheu vasculitis w rheumatoid arthritis of unsp shoulder
M0522	ICD10	Rheumatoid vasculitis with rheumatoid arthritis of elbow
M05221	ICD10	Rheumatoid vasculitis w rheumatoid arthritis of right elbow
M05222	ICD10	Rheumatoid vasculitis w rheumatoid arthritis of left elbow
M05229	ICD10	Rheumatoid vasculitis w rheumatoid arthritis of unsp elbow
M0523	ICD10	Rheumatoid vasculitis with rheumatoid arthritis of wrist
M05231	ICD10	Rheumatoid vasculitis w rheumatoid arthritis of right wrist
M05232	ICD10	Rheumatoid vasculitis w rheumatoid arthritis of left wrist
M05239	ICD10	Rheumatoid vasculitis w rheumatoid arthritis of unsp wrist

DIAG_FMT	CODE_TYPE	DIAG_DESC
M0524	ICD10	Rheumatoid vasculitis with rheumatoid arthritis of hand
M05241	ICD10	Rheumatoid vasculitis w rheumatoid arthritis of right hand
M05242	ICD10	Rheumatoid vasculitis with rheumatoid arthritis of left hand
M05249	ICD10	Rheumatoid vasculitis with rheumatoid arthritis of unsp hand
M0525	ICD10	Rheumatoid vasculitis with rheumatoid arthritis of hip
M05251	ICD10	Rheumatoid vasculitis with rheumatoid arthritis of right hip
M05252	ICD10	Rheumatoid vasculitis with rheumatoid arthritis of left hip
M05259	ICD10	Rheumatoid vasculitis with rheumatoid arthritis of unsp hip
M0526	ICD10	Rheumatoid vasculitis with rheumatoid arthritis of knee
M05261	ICD10	Rheumatoid vasculitis w rheumatoid arthritis of right knee
M05262	ICD10	Rheumatoid vasculitis with rheumatoid arthritis of left knee
M05269	ICD10	Rheumatoid vasculitis with rheumatoid arthritis of unsp knee
M0527	ICD10	Rheumatoid vasculitis w rheumatoid arthritis of ank/ft
M05271	ICD10	Rheumatoid vasculitis w rheumatoid arthritis of right ank/ft
M05272	ICD10	Rheumatoid vasculitis w rheumatoid arthritis of left ank/ft
M05279	ICD10	Rheumatoid vasculitis w rheumatoid arthritis of unsp ank/ft
M0529	ICD10	Rheumatoid vasculitis w rheumatoid arthritis mult site
M053	ICD10	Rheumatoid heart disease with rheumatoid arthritis
M0530	ICD10	Rheumatoid heart disease w rheumatoid arthritis of unsp site
M0531	ICD10	Rheumatoid heart disease w rheumatoid arthritis of shoulder
M05311	ICD10	Rheu heart disease w rheumatoid arthritis of r shoulder
M05312	ICD10	Rheu heart disease w rheumatoid arthritis of l shoulder
M05319	ICD10	Rheu heart disease w rheumatoid arthritis of unsp shoulder
M0532	ICD10	Rheumatoid heart disease with rheumatoid arthritis of elbow
M05321	ICD10	Rheumatoid heart disease w rheumatoid arthritis of r elbow
M05322	ICD10	Rheumatoid heart disease w rheumatoid arthritis of l elbow
M05329	ICD10	Rheu heart disease w rheumatoid arthritis of unsp elbow
M0533	ICD10	Rheumatoid heart disease with rheumatoid arthritis of wrist
M05331	ICD10	Rheumatoid heart disease w rheumatoid arthritis of r wrist
M05332	ICD10	Rheumatoid heart disease w rheumatoid arthritis of l wrist
M05339	ICD10	Rheu heart disease w rheumatoid arthritis of unsp wrist
M0534	ICD10	Rheumatoid heart disease with rheumatoid arthritis of hand
M05341	ICD10	Rheu heart disease w rheumatoid arthritis of right hand
M05342	ICD10	Rheumatoid heart disease w rheumatoid arthritis of left hand
M05349	ICD10	Rheumatoid heart disease w rheumatoid arthritis of unsp hand
M0535	ICD10	Rheumatoid heart disease with rheumatoid arthritis of hip
M05351	ICD10	Rheumatoid heart disease w rheumatoid arthritis of right hip
M05352	ICD10	Rheumatoid heart disease w rheumatoid arthritis of left hip
M05359	ICD10	Rheumatoid heart disease w rheumatoid arthritis of unsp hip
M0536	ICD10	Rheumatoid heart disease with rheumatoid arthritis of knee
M05361	ICD10	Rheu heart disease w rheumatoid arthritis of right knee
M05362	ICD10	Rheumatoid heart disease w rheumatoid arthritis of left knee
M05369	ICD10	Rheumatoid heart disease w rheumatoid arthritis of unsp knee
M0537	ICD10	Rheumatoid heart disease w rheumatoid arthritis of ank/ft
M05371	ICD10	Rheu heart disease w rheumatoid arthritis of right ank/ft
M05372	ICD10	Rheu heart disease w rheumatoid arthritis of left ank/ft

DIAG_FMT	CODE_TYPE	DIAG_DESC
M05379	ICD10	Rheu heart disease w rheumatoid arthritis of unsp ank/ft
M0539	ICD10	Rheumatoid heart disease w rheumatoid arthritis mult site
M054	ICD10	Rheumatoid myopathy with rheumatoid arthritis
M0540	ICD10	Rheumatoid myopathy with rheumatoid arthritis of unsp site
M0541	ICD10	Rheumatoid myopathy with rheumatoid arthritis of shoulder
M05411	ICD10	Rheumatoid myopathy w rheumatoid arthritis of right shoulder
M05412	ICD10	Rheumatoid myopathy w rheumatoid arthritis of left shoulder
M05419	ICD10	Rheumatoid myopathy w rheumatoid arthritis of unsp shoulder
M0542	ICD10	Rheumatoid myopathy with rheumatoid arthritis of elbow
M05421	ICD10	Rheumatoid myopathy with rheumatoid arthritis of right elbow
M05422	ICD10	Rheumatoid myopathy with rheumatoid arthritis of left elbow
M05429	ICD10	Rheumatoid myopathy with rheumatoid arthritis of unsp elbow
M0543	ICD10	Rheumatoid myopathy with rheumatoid arthritis of wrist
M05431	ICD10	Rheumatoid myopathy with rheumatoid arthritis of right wrist
M05432	ICD10	Rheumatoid myopathy with rheumatoid arthritis of left wrist
M05439	ICD10	Rheumatoid myopathy with rheumatoid arthritis of unsp wrist
M0544	ICD10	Rheumatoid myopathy with rheumatoid arthritis of hand
M05441	ICD10	Rheumatoid myopathy with rheumatoid arthritis of right hand
M05442	ICD10	Rheumatoid myopathy with rheumatoid arthritis of left hand
M05449	ICD10	Rheumatoid myopathy with rheumatoid arthritis of unsp hand
M0545	ICD10	Rheumatoid myopathy with rheumatoid arthritis of hip
M05451	ICD10	Rheumatoid myopathy with rheumatoid arthritis of right hip
M05452	ICD10	Rheumatoid myopathy with rheumatoid arthritis of left hip
M05459	ICD10	Rheumatoid myopathy with rheumatoid arthritis of unsp hip
M0546	ICD10	Rheumatoid myopathy with rheumatoid arthritis of knee
M05461	ICD10	Rheumatoid myopathy with rheumatoid arthritis of right knee
M05462	ICD10	Rheumatoid myopathy with rheumatoid arthritis of left knee
M05469	ICD10	Rheumatoid myopathy with rheumatoid arthritis of unsp knee
M0547	ICD10	Rheumatoid myopathy w rheumatoid arthritis of ankle and foot
M05471	ICD10	Rheumatoid myopathy w rheumatoid arthritis of right ank/ft
M05472	ICD10	Rheumatoid myopathy w rheumatoid arthritis of left ank/ft
M05479	ICD10	Rheumatoid myopathy w rheumatoid arthritis of unsp ank/ft
M0549	ICD10	Rheumatoid myopathy w rheumatoid arthritis of multiple sites
M055	ICD10	Rheumatoid polyneuropathy with rheumatoid arthritis
M0550	ICD10	Rheumatoid polyneurop w rheumatoid arthritis of unsp site
M0551	ICD10	Rheumatoid polyneuropathy w rheumatoid arthritis of shoulder
M05511	ICD10	Rheumatoid polyneurop w rheumatoid arthritis of r shoulder
M05512	ICD10	Rheumatoid polyneurop w rheumatoid arthritis of l shoulder
M05519	ICD10	Rheu polyneurop w rheumatoid arthritis of unsp shoulder
M0552	ICD10	Rheumatoid polyneuropathy with rheumatoid arthritis of elbow
M05521	ICD10	Rheumatoid polyneurop w rheumatoid arthritis of right elbow
M05522	ICD10	Rheumatoid polyneurop w rheumatoid arthritis of left elbow
M05529	ICD10	Rheumatoid polyneurop w rheumatoid arthritis of unsp elbow
M0553	ICD10	Rheumatoid polyneuropathy with rheumatoid arthritis of wrist
M05531	ICD10	Rheumatoid polyneurop w rheumatoid arthritis of right wrist
M05532	ICD10	Rheumatoid polyneurop w rheumatoid arthritis of left wrist

DIAG_FMT	CODE_TYPE	DIAG_DESC
M05539	ICD10	Rheumatoid polyneurop w rheumatoid arthritis of unsp wrist
M0554	ICD10	Rheumatoid polyneuropathy with rheumatoid arthritis of hand
M05541	ICD10	Rheumatoid polyneurop w rheumatoid arthritis of right hand
M05542	ICD10	Rheumatoid polyneurop w rheumatoid arthritis of left hand
M05549	ICD10	Rheumatoid polyneurop w rheumatoid arthritis of unsp hand
M0555	ICD10	Rheumatoid polyneuropathy with rheumatoid arthritis of hip
M05551	ICD10	Rheumatoid polyneurop w rheumatoid arthritis of right hip
M05552	ICD10	Rheumatoid polyneuropathy w rheumatoid arthritis of left hip
M05559	ICD10	Rheumatoid polyneuropathy w rheumatoid arthritis of unsp hip
M0556	ICD10	Rheumatoid polyneuropathy with rheumatoid arthritis of knee
M05561	ICD10	Rheumatoid polyneurop w rheumatoid arthritis of right knee
M05562	ICD10	Rheumatoid polyneurop w rheumatoid arthritis of left knee
M05569	ICD10	Rheumatoid polyneurop w rheumatoid arthritis of unsp knee
M0557	ICD10	Rheumatoid polyneuropathy w rheumatoid arthritis of ank/ft
M05571	ICD10	Rheumatoid polyneurop w rheumatoid arthritis of right ank/ft
M05572	ICD10	Rheumatoid polyneurop w rheumatoid arthritis of left ank/ft
M05579	ICD10	Rheumatoid polyneurop w rheumatoid arthritis of unsp ank/ft
M0559	ICD10	Rheumatoid polyneuropathy w rheumatoid arthritis mult site
M056	ICD10	Rheumatoid arthritis w involvement of oth organs and systems
M0560	ICD10	Rheu arthritis of unsp site w involv of organs and systems
M0561	ICD10	Rheu arthritis of shoulder w involv of organs and systems
M05611	ICD10	Rheu arthritis of r shoulder w involv of organs and systems
M05612	ICD10	Rheu arthritis of l shoulder w involv of organs and systems
M05619	ICD10	Rheu arthrit of unsp shoulder w involv of organs and systems
M0562	ICD10	Rheumatoid arthritis of elbow w involv of organs and systems
M05621	ICD10	Rheu arthritis of r elbow w involv of organs and systems
M05622	ICD10	Rheu arthritis of l elbow w involv of organs and systems
M05629	ICD10	Rheu arthritis of unsp elbow w involv of organs and systems
M0563	ICD10	Rheumatoid arthritis of wrist w involv of organs and systems
M05631	ICD10	Rheu arthritis of r wrist w involv of organs and systems
M05632	ICD10	Rheu arthritis of l wrist w involv of organs and systems
M05639	ICD10	Rheu arthritis of unsp wrist w involv of organs and systems
M0564	ICD10	Rheumatoid arthritis of hand w involv of organs and systems
M05641	ICD10	Rheu arthritis of right-hand w involv of organs and systems
M05642	ICD10	Rheu arthritis of left-hand w involv of organs and systems
M05649	ICD10	Rheu arthritis of unsp hand w involv of organs and systems
M0565	ICD10	Rheumatoid arthritis of hip w involv of organs and systems
M05651	ICD10	Rheu arthritis of right hip w involv of organs and systems
M05652	ICD10	Rheu arthritis of left hip w involv of organs and systems
M05659	ICD10	Rheu arthritis of unsp hip w involv of organs and systems
M0566	ICD10	Rheumatoid arthritis of knee w involv of organs and systems
M05661	ICD10	Rheu arthritis of right knee w involv of organs and systems
M05662	ICD10	Rheu arthritis of left knee w involv of organs and systems
M05669	ICD10	Rheu arthritis of unsp knee w involv of organs and systems
M0567	ICD10	Rheu arthritis of ank/ft w involv of organs and systems
M05671	ICD10	Rheu arthrit of right ank/ft w involv of organs and systems

DIAG_FMT	CODE_TYPE	DIAG_DESC
M05672	ICD10	Rheu arthritis of left ank/ft w involv of organs and systems
M05679	ICD10	Rheu arthritis of unsp ank/ft w involv of organs and systems
M0569	ICD10	Rheu arthritis mult site w involv of organs and systems
M057	ICD10	Rheumatoid arthritis w rheumatoid factor w/o org/sys involv
M0570	ICD10	Rheu arthritis w rheu factor of unsp site w/o org/sys involv
M0571	ICD10	Rheu arthritis w rheu factor of shoulder w/o org/sys involv
M05711	ICD10	Rheu arthrit w rheu factor of r shoulder w/o org/sys involv
M05712	ICD10	Rheu arthrit w rheu factor of l shoulder w/o org/sys involv
M05719	ICD10	Rheu arthrit w rheu factor of unsp shldr w/o org/sys involv
M0572	ICD10	Rheu arthritis w rheu factor of elbow w/o org/sys involv
M05721	ICD10	Rheu arthritis w rheu factor of r elbow w/o org/sys involv
M05722	ICD10	Rheu arthritis w rheu factor of l elbow w/o org/sys involv
M05729	ICD10	Rheu arthrit w rheu factor of unsp elbow w/o org/sys involv
M0573	ICD10	Rheu arthritis w rheu factor of wrist w/o org/sys involv
M05731	ICD10	Rheu arthritis w rheu factor of r wrist w/o org/sys involv
M05732	ICD10	Rheu arthritis w rheu factor of l wrist w/o org/sys involv
M05739	ICD10	Rheu arthrit w rheu factor of unsp wrist w/o org/sys involv
M0574	ICD10	Rheu arthritis w rheu factor of hand w/o org/sys involv
M05741	ICD10	Rheu arthritis w rheu factor of r hand w/o org/sys involv
M05742	ICD10	Rheu arthritis w rheu factor of left-hand w/o org/sys involv
M05749	ICD10	Rheu arthritis w rheu factor of unsp hand w/o org/sys involv
M0575	ICD10	Rheu arthritis w rheumatoid factor of hip w/o org/sys involv
M05751	ICD10	Rheu arthritis w rheu factor of right hip w/o org/sys involv
M05752	ICD10	Rheu arthritis w rheu factor of left hip w/o org/sys involv
M05759	ICD10	Rheu arthritis w rheu factor of unsp hip w/o org/sys involv
M0576	ICD10	Rheu arthritis w rheu factor of knee w/o org/sys involv
M05761	ICD10	Rheu arthritis w rheu factor of r knee w/o org/sys involv
M05762	ICD10	Rheu arthritis w rheu factor of left knee w/o org/sys involv
M05769	ICD10	Rheu arthritis w rheu factor of unsp knee w/o org/sys involv
M0577	ICD10	Rheu arthritis w rheu factor of ank/ft w/o org/sys involv
M05771	ICD10	Rheu arthrit w rheu fctr of right ank/ft w/o org/sys involv
M05772	ICD10	Rheu arthrit w rheu factor of left ank/ft w/o org/sys involv
M05779	ICD10	Rheu arthrit w rheu factor of unsp ank/ft w/o org/sys involv
M0579	ICD10	Rheu arthritis w rheu factor mult site w/o org/sys involv
M058	ICD10	Other rheumatoid arthritis with rheumatoid factor
M0580	ICD10	Oth rheumatoid arthritis with rheumatoid factor of unsp site
M0581	ICD10	Oth rheumatoid arthritis with rheumatoid factor of shoulder
M05811	ICD10	Oth rheumatoid arthritis w rheumatoid factor of r shoulder
M05812	ICD10	Oth rheumatoid arthritis w rheumatoid factor of l shoulder
M05819	ICD10	Oth rheu arthritis w rheumatoid factor of unsp shoulder
M0582	ICD10	Other rheumatoid arthritis with rheumatoid factor of elbow
M05821	ICD10	Oth rheumatoid arthritis w rheumatoid factor of right elbow
M05822	ICD10	Oth rheumatoid arthritis w rheumatoid factor of left elbow
M05829	ICD10	Oth rheumatoid arthritis w rheumatoid factor of unsp elbow
M0583	ICD10	Other rheumatoid arthritis with rheumatoid factor of wrist
M05831	ICD10	Oth rheumatoid arthritis w rheumatoid factor of right wrist

DIAG_FMT	CODE_TYPE	DIAG_DESC
M05832	ICD10	Oth rheumatoid arthritis w rheumatoid factor of left wrist
M05839	ICD10	Oth rheumatoid arthritis w rheumatoid factor of unsp wrist
M0584	ICD10	Other rheumatoid arthritis with rheumatoid factor of hand
M05841	ICD10	Oth rheumatoid arthritis w rheumatoid factor of right hand
M05842	ICD10	Oth rheumatoid arthritis with rheumatoid factor of left hand
M05849	ICD10	Oth rheumatoid arthritis with rheumatoid factor of unsp hand
M0585	ICD10	Other rheumatoid arthritis with rheumatoid factor of hip
M05851	ICD10	Oth rheumatoid arthritis with rheumatoid factor of right hip
M05852	ICD10	Oth rheumatoid arthritis with rheumatoid factor of left hip
M05859	ICD10	Oth rheumatoid arthritis with rheumatoid factor of unsp hip
M0586	ICD10	Other rheumatoid arthritis with rheumatoid factor of knee
M05861	ICD10	Oth rheumatoid arthritis w rheumatoid factor of right knee
M05862	ICD10	Oth rheumatoid arthritis with rheumatoid factor of left knee
M05869	ICD10	Oth rheumatoid arthritis with rheumatoid factor of unsp knee
M0587	ICD10	Oth rheumatoid arthritis w rheumatoid factor of ank/ft
M05871	ICD10	Oth rheumatoid arthritis w rheumatoid factor of right ank/ft
M05872	ICD10	Oth rheumatoid arthritis w rheumatoid factor of left ank/ft
M05879	ICD10	Oth rheumatoid arthritis w rheumatoid factor of unsp ank/ft
M0589	ICD10	Oth rheumatoid arthritis w rheumatoid factor mult site
M059	ICD10	Rheumatoid arthritis with rheumatoid factor, unspecified
M06	ICD10	Other rheumatoid arthritis
M060	ICD10	Rheumatoid arthritis without rheumatoid factor
M0600	ICD10	Rheumatoid arthritis without rheumatoid factor, unsp site
M0601	ICD10	Rheumatoid arthritis without rheumatoid factor, shoulder
M06011	ICD10	Rheumatoid arthritis w/o rheumatoid factor, right shoulder
M06012	ICD10	Rheumatoid arthritis w/o rheumatoid factor, left shoulder
M06019	ICD10	Rheumatoid arthritis w/o rheumatoid factor, unsp shoulder
M0602	ICD10	Rheumatoid arthritis without rheumatoid factor, elbow
M06021	ICD10	Rheumatoid arthritis without rheumatoid factor, right elbow
M06022	ICD10	Rheumatoid arthritis without rheumatoid factor, left elbow
M06029	ICD10	Rheumatoid arthritis without rheumatoid factor, unsp elbow
M0603	ICD10	Rheumatoid arthritis without rheumatoid factor, wrist
M06031	ICD10	Rheumatoid arthritis without rheumatoid factor, right wrist
M06032	ICD10	Rheumatoid arthritis without rheumatoid factor, left wrist
M06039	ICD10	Rheumatoid arthritis without rheumatoid factor, unsp wrist
M0604	ICD10	Rheumatoid arthritis without rheumatoid factor, hand
M06041	ICD10	Rheumatoid arthritis without rheumatoid factor, right hand
M06042	ICD10	Rheumatoid arthritis without rheumatoid factor, left hand
M06049	ICD10	Rheumatoid arthritis without rheumatoid factor, unsp hand
M0605	ICD10	Rheumatoid arthritis without rheumatoid factor, hip
M06051	ICD10	Rheumatoid arthritis without rheumatoid factor, right hip
M06052	ICD10	Rheumatoid arthritis without rheumatoid factor, left hip
M06059	ICD10	Rheumatoid arthritis without rheumatoid factor, unsp hip
M0606	ICD10	Rheumatoid arthritis without rheumatoid factor, knee
M06061	ICD10	Rheumatoid arthritis without rheumatoid factor, right knee
M06062	ICD10	Rheumatoid arthritis without rheumatoid factor, left knee

DIAG_FMT	CODE_TYPE	DIAG_DESC
M06069	ICD10	Rheumatoid arthritis without rheumatoid factor, unsp knee
M0607	ICD10	Rheumatoid arthritis w/o rheumatoid factor, ankle, and foot
M06071	ICD10	Rheumatoid arthritis w/o rheumatoid factor, right ank/ft
M06072	ICD10	Rheumatoid arthritis w/o rheumatoid factor, left ank/ft
M06079	ICD10	Rheumatoid arthritis w/o rheumatoid factor, unsp ank/ft
M0608	ICD10	Rheumatoid arthritis without rheumatoid factor, vertebrae
M0609	ICD10	Rheumatoid arthritis w/o rheumatoid factor, multiple sites
M061	ICD10	Adult-onset Still's disease
M062	ICD10	Rheumatoid bursitis
M0620	ICD10	Rheumatoid bursitis, unspecified site
M0621	ICD10	Rheumatoid bursitis, shoulder
M06211	ICD10	Rheumatoid bursitis, right shoulder
M06212	ICD10	Rheumatoid bursitis, left shoulder
M06219	ICD10	Rheumatoid bursitis, unspecified shoulder
M0622	ICD10	Rheumatoid bursitis, elbow
M06221	ICD10	Rheumatoid bursitis, right elbow
M06222	ICD10	Rheumatoid bursitis, left elbow
M06229	ICD10	Rheumatoid bursitis, unspecified elbow
M0623	ICD10	Rheumatoid bursitis, wrist
M06231	ICD10	Rheumatoid bursitis, right wrist
M06232	ICD10	Rheumatoid bursitis, left wrist
M06239	ICD10	Rheumatoid bursitis, unspecified wrist
M0624	ICD10	Rheumatoid bursitis, hand
M06241	ICD10	Rheumatoid bursitis, right hand
M06242	ICD10	Rheumatoid bursitis, left hand
M06249	ICD10	Rheumatoid bursitis, unspecified hand
M0625	ICD10	Rheumatoid bursitis, hip
M06251	ICD10	Rheumatoid bursitis, right hip
M06252	ICD10	Rheumatoid bursitis, left hip
M06259	ICD10	Rheumatoid bursitis, unspecified hip
M0626	ICD10	Rheumatoid bursitis, knee
M06261	ICD10	Rheumatoid bursitis, right knee
M06262	ICD10	Rheumatoid bursitis, left knee
M06269	ICD10	Rheumatoid bursitis, unspecified knee
M0627	ICD10	Rheumatoid bursitis, ankle, and foot
M06271	ICD10	Rheumatoid bursitis, right ankle, and foot
M06272	ICD10	Rheumatoid bursitis, left ankle and foot
M06279	ICD10	Rheumatoid bursitis, unspecified ankle, and foot
M0628	ICD10	Rheumatoid bursitis, vertebrae
M0629	ICD10	Rheumatoid bursitis, multiple sites
M063	ICD10	Rheumatoid nodule
M0630	ICD10	Rheumatoid nodule, unspecified site
M0631	ICD10	Rheumatoid nodule, shoulder
M06311	ICD10	Rheumatoid nodule, right shoulder
M06312	ICD10	Rheumatoid nodule, left shoulder
M06319	ICD10	Rheumatoid nodule, unspecified shoulder

DIAG_FMT	CODE_TYPE	DIAG_DESC
M0632	ICD10	Rheumatoid nodule, elbow
M06321	ICD10	Rheumatoid nodule, right elbow
M06322	ICD10	Rheumatoid nodule, left elbow
M06329	ICD10	Rheumatoid nodule, unspecified elbow
M0633	ICD10	Rheumatoid nodule, wrist
M06331	ICD10	Rheumatoid nodule, right wrist
M06332	ICD10	Rheumatoid nodule, left wrist
M06339	ICD10	Rheumatoid nodule, unspecified wrist
M0634	ICD10	Rheumatoid nodule, hand
M06341	ICD10	Rheumatoid nodule, right hand
M06342	ICD10	Rheumatoid nodule, left hand
M06349	ICD10	Rheumatoid nodule, unspecified hand
M0635	ICD10	Rheumatoid nodule, hip
M06351	ICD10	Rheumatoid nodule, right hip
M06352	ICD10	Rheumatoid nodule, left hip
M06359	ICD10	Rheumatoid nodule, unspecified hip
M0636	ICD10	Rheumatoid nodule, knee
M06361	ICD10	Rheumatoid nodule, right knee
M06362	ICD10	Rheumatoid nodule, left knee
M06369	ICD10	Rheumatoid nodule, unspecified knee
M0637	ICD10	Rheumatoid nodule, ankle, and foot
M06371	ICD10	Rheumatoid nodule, right ankle, and foot
M06372	ICD10	Rheumatoid nodule, left ankle and foot
M06379	ICD10	Rheumatoid nodule, unspecified ankle, and foot
M0638	ICD10	Rheumatoid nodule, vertebrae
M0639	ICD10	Rheumatoid nodule, multiple sites
M064	ICD10	Inflammatory polyarthropathy
M068	ICD10	Other specified rheumatoid arthritis
M0680	ICD10	Other specified rheumatoid arthritis, unspecified site
M0681	ICD10	Other specified rheumatoid arthritis, shoulder
M06811	ICD10	Other specified rheumatoid arthritis, right shoulder
M06812	ICD10	Other specified rheumatoid arthritis, left shoulder
M06819	ICD10	Other specified rheumatoid arthritis, unspecified shoulder
M0682	ICD10	Other specified rheumatoid arthritis, elbow
M06821	ICD10	Other specified rheumatoid arthritis, right elbow
M06822	ICD10	Other specified rheumatoid arthritis, left elbow
M06829	ICD10	Other specified rheumatoid arthritis, unspecified elbow
M0683	ICD10	Other specified rheumatoid arthritis, wrist
M06831	ICD10	Other specified rheumatoid arthritis, right wrist
M06832	ICD10	Other specified rheumatoid arthritis, left wrist
M06839	ICD10	Other specified rheumatoid arthritis, unspecified wrist
M0684	ICD10	Other specified rheumatoid arthritis, hand
M06841	ICD10	Other specified rheumatoid arthritis, right hand
M06842	ICD10	Other specified rheumatoid arthritis, left hand
M06849	ICD10	Other specified rheumatoid arthritis, unspecified hand
M0685	ICD10	Other specified rheumatoid arthritis, hip

DIAG_FMT	CODE_TYPE	DIAG_DESC
M06851	ICD10	Other specified rheumatoid arthritis, right hip
M06852	ICD10	Other specified rheumatoid arthritis, left hip
M06859	ICD10	Other specified rheumatoid arthritis, unspecified hip
M0686	ICD10	Other specified rheumatoid arthritis, knee
M06861	ICD10	Other specified rheumatoid arthritis, right knee
M06862	ICD10	Other specified rheumatoid arthritis, left knee
M06869	ICD10	Other specified rheumatoid arthritis, unspecified knee
M0687	ICD10	Other specified rheumatoid arthritis, ankle, and foot
M06871	ICD10	Other specified rheumatoid arthritis, right ankle, and foot
M06872	ICD10	Other specified rheumatoid arthritis, left ankle and foot
M06879	ICD10	Oth rheumatoid arthritis, unspecified ankle, and foot
M0688	ICD10	Other specified rheumatoid arthritis, vertebrae
M0689	ICD10	Other specified rheumatoid arthritis, multiple sites
M069	ICD10	Rheumatoid arthritis, unspecified
M08	ICD10	Juvenile arthritis
M080	ICD10	Unspecified juvenile rheumatoid arthritis
M0800	ICD10	Unsp juvenile rheumatoid arthritis of unspecified site
M0801	ICD10	Unspecified juvenile rheumatoid arthritis, shoulder
M08011	ICD10	Unspecified juvenile rheumatoid arthritis, right shoulder
M08012	ICD10	Unspecified juvenile rheumatoid arthritis, left shoulder
M08019	ICD10	Unsp juvenile rheumatoid arthritis, unspecified shoulder
M0802	ICD10	Unspecified juvenile rheumatoid arthritis of elbow
M08021	ICD10	Unspecified juvenile rheumatoid arthritis, right elbow
M08022	ICD10	Unspecified juvenile rheumatoid arthritis, left elbow
M08029	ICD10	Unspecified juvenile rheumatoid arthritis, unspecified elbow
M0803	ICD10	Unspecified juvenile rheumatoid arthritis, wrist
M08031	ICD10	Unspecified juvenile rheumatoid arthritis, right wrist
M08032	ICD10	Unspecified juvenile rheumatoid arthritis, left wrist
M08039	ICD10	Unspecified juvenile rheumatoid arthritis, unspecified wrist
M0804	ICD10	Unspecified juvenile rheumatoid arthritis, hand
M08041	ICD10	Unspecified juvenile rheumatoid arthritis, right hand
M08042	ICD10	Unspecified juvenile rheumatoid arthritis, left hand
M08049	ICD10	Unspecified juvenile rheumatoid arthritis, unspecified hand
M0805	ICD10	Unspecified juvenile rheumatoid arthritis, hip
M08051	ICD10	Unspecified juvenile rheumatoid arthritis, right hip
M08052	ICD10	Unspecified juvenile rheumatoid arthritis, left hip
M08059	ICD10	Unspecified juvenile rheumatoid arthritis, unspecified hip
M0806	ICD10	Unspecified juvenile rheumatoid arthritis, knee
M08061	ICD10	Unspecified juvenile rheumatoid arthritis, right knee
M08062	ICD10	Unspecified juvenile rheumatoid arthritis, left knee
M08069	ICD10	Unspecified juvenile rheumatoid arthritis, unspecified knee
M0807	ICD10	Unspecified juvenile rheumatoid arthritis, ankle, and foot
M08071	ICD10	Unsp juvenile rheumatoid arthritis, right ankle, and foot
M08072	ICD10	Unsp juvenile rheumatoid arthritis, left ankle and foot
M08079	ICD10	Unsp juvenile rheumatoid arthritis, unsp ankle and foot
M0808	ICD10	Unspecified juvenile rheumatoid arthritis, vertebrae

DIAG_FMT	CODE_TYPE	DIAG_DESC
M0809	ICD10	Unspecified juvenile rheumatoid arthritis, multiple sites
M081	ICD10	Juvenile ankylosing spondylitis
M082	ICD10	Juvenile rheumatoid arthritis with systemic onset
M0820	ICD10	Juvenile rheumatoid arthritis with systemic onset, unsp site
M0821	ICD10	Juvenile rheumatoid arthritis with systemic onset, shoulder
M08211	ICD10	Juvenile rheumatoid arthritis w systemic onset, r shoulder
M08212	ICD10	Juvenile rheumatoid arthritis w systemic onset, l shoulder
M08219	ICD10	Juvenile rheu arthritis w systemic onset, unsp shoulder
M0822	ICD10	Juvenile rheumatoid arthritis with systemic onset, elbow
M08221	ICD10	Juvenile rheumatoid arthritis w systemic onset, right elbow
M08222	ICD10	Juvenile rheumatoid arthritis w systemic onset, left elbow
M08229	ICD10	Juvenile rheumatoid arthritis w systemic onset, unsp elbow
M0823	ICD10	Juvenile rheumatoid arthritis with systemic onset, wrist
M08231	ICD10	Juvenile rheumatoid arthritis w systemic onset, right wrist
M08232	ICD10	Juvenile rheumatoid arthritis w systemic onset, left wrist
M08239	ICD10	Juvenile rheumatoid arthritis w systemic onset, unsp wrist
M0824	ICD10	Juvenile rheumatoid arthritis with systemic onset, hand
M08241	ICD10	Juvenile rheumatoid arthritis w systemic onset, right hand
M08242	ICD10	Juvenile rheumatoid arthritis with systemic onset, left hand
M08249	ICD10	Juvenile rheumatoid arthritis with systemic onset, unsp hand
M0825	ICD10	Juvenile rheumatoid arthritis with systemic onset, hip
M08251	ICD10	Juvenile rheumatoid arthritis with systemic onset, right hip
M08252	ICD10	Juvenile rheumatoid arthritis with systemic onset, left hip
M08259	ICD10	Juvenile rheumatoid arthritis with systemic onset, unsp hip
M0826	ICD10	Juvenile rheumatoid arthritis with systemic onset, knee
M08261	ICD10	Juvenile rheumatoid arthritis w systemic onset, right knee
M08262	ICD10	Juvenile rheumatoid arthritis with systemic onset, left knee
M08269	ICD10	Juvenile rheumatoid arthritis with systemic onset, unsp knee
M0827	ICD10	Juvenile rheumatoid arthritis w systemic onset, ank/ft
M08271	ICD10	Juvenile rheumatoid arthritis w systemic onset, right ank/ft
M08272	ICD10	Juvenile rheumatoid arthritis w systemic onset, left ank/ft
M08279	ICD10	Juvenile rheumatoid arthritis w systemic onset, unsp ank/ft
M0828	ICD10	Juvenile rheumatoid arthritis with systemic onset, vertebrae
M0829	ICD10	Juvenile rheu arthritis w systemic onset, multiple sites
M083	ICD10	Juvenile rheumatoid polyarthritis (seronegative)
M084	ICD10	Pauciarticular juvenile rheumatoid arthritis
M0840	ICD10	Pauciarticular juvenile rheumatoid arthritis, unsp site
M0841	ICD10	Pauciarticular juvenile rheumatoid arthritis, shoulder
M08411	ICD10	Pauciarticular juvenile rheumatoid arthritis, right shoulder
M08412	ICD10	Pauciarticular juvenile rheumatoid arthritis, left shoulder
M08419	ICD10	Pauciarticular juvenile rheumatoid arthritis, unsp shoulder
M0842	ICD10	Pauciarticular juvenile rheumatoid arthritis, elbow
M08421	ICD10	Pauciarticular juvenile rheumatoid arthritis, right elbow
M08422	ICD10	Pauciarticular juvenile rheumatoid arthritis, left elbow
M08429	ICD10	Pauciarticular juvenile rheumatoid arthritis, unsp elbow
M0843	ICD10	Pauciarticular juvenile rheumatoid arthritis, wrist

DIAG_FMT	CODE_TYPE	DIAG_DESC
M08431	ICD10	Pauciarticular juvenile rheumatoid arthritis, right wrist
M08432	ICD10	Pauciarticular juvenile rheumatoid arthritis, left wrist
M08439	ICD10	Pauciarticular juvenile rheumatoid arthritis, unsp wrist
M0844	ICD10	Pauciarticular juvenile rheumatoid arthritis, hand
M08441	ICD10	Pauciarticular juvenile rheumatoid arthritis, right hand
M08442	ICD10	Pauciarticular juvenile rheumatoid arthritis, left hand
M08449	ICD10	Pauciarticular juvenile rheumatoid arthritis, unsp hand
M0845	ICD10	Pauciarticular juvenile rheumatoid arthritis, hip
M08451	ICD10	Pauciarticular juvenile rheumatoid arthritis, right hip
M08452	ICD10	Pauciarticular juvenile rheumatoid arthritis, left hip
M08459	ICD10	Pauciarticular juvenile rheumatoid arthritis, unsp hip
M0846	ICD10	Pauciarticular juvenile rheumatoid arthritis, knee
M08461	ICD10	Pauciarticular juvenile rheumatoid arthritis, right knee
M08462	ICD10	Pauciarticular juvenile rheumatoid arthritis, left knee
M08469	ICD10	Pauciarticular juvenile rheumatoid arthritis, unsp knee
M0847	ICD10	Pauciarticular juvenile rheumatoid arthritis, ankle, and foot
M08471	ICD10	Pauciarticular juvenile rheumatoid arthritis, right ank/ft
M08472	ICD10	Pauciarticular juvenile rheumatoid arthritis, left ank/ft
M08479	ICD10	Pauciarticular juvenile rheumatoid arthritis, unsp ank/ft
M0848	ICD10	Pauciarticular juvenile rheumatoid arthritis, vertebrae
M088	ICD10	Other juvenile arthritis
M0880	ICD10	Other juvenile arthritis, unspecified site
M0881	ICD10	Other juvenile arthritis, shoulder
M08811	ICD10	Other juvenile arthritis, right shoulder
M08812	ICD10	Other juvenile arthritis, left shoulder
M08819	ICD10	Other juvenile arthritis, unspecified shoulder
M0882	ICD10	Other juvenile arthritis, elbow
M08821	ICD10	Other juvenile arthritis, right elbow
M08822	ICD10	Other juvenile arthritis, left elbow
M08829	ICD10	Other juvenile arthritis, unspecified elbow
M0883	ICD10	Other juvenile arthritis, wrist
M08831	ICD10	Other juvenile arthritis, right wrist
M08832	ICD10	Other juvenile arthritis, left wrist
M08839	ICD10	Other juvenile arthritis, unspecified wrist
M0884	ICD10	Other juvenile arthritis, hand
M08841	ICD10	Other juvenile arthritis, right hand
M08842	ICD10	Other juvenile arthritis, left hand
M08849	ICD10	Other juvenile arthritis, unspecified hand
M0885	ICD10	Other juvenile arthritis, hip
M08851	ICD10	Other juvenile arthritis, right hip
M08852	ICD10	Other juvenile arthritis, left hip
M08859	ICD10	Other juvenile arthritis, unspecified hip
M0886	ICD10	Other juvenile arthritis, knee
M08861	ICD10	Other juvenile arthritis, right knee
M08862	ICD10	Other juvenile arthritis, left knee
M08869	ICD10	Other juvenile arthritis, unspecified knee

DIAG_FMT	CODE_TYPE	DIAG_DESC
M0887	ICD10	Other juvenile arthritis, ankle, and foot
M08871	ICD10	Other juvenile arthritis, right ankle, and foot
M08872	ICD10	Other juvenile arthritis, left ankle and foot
M08879	ICD10	Other juvenile arthritis, unspecified ankle, and foot
M0888	ICD10	Other juvenile arthritis, other specified site
M0889	ICD10	Other juvenile arthritis, multiple sites
M089	ICD10	Juvenile arthritis, unspecified
M0890	ICD10	Juvenile arthritis, unspecified, unspecified site
M0891	ICD10	Juvenile arthritis, unspecified, shoulder
M08911	ICD10	Juvenile arthritis, unspecified, right shoulder
M08912	ICD10	Juvenile arthritis, unspecified, left shoulder
M08919	ICD10	Juvenile arthritis, unspecified, unspecified shoulder
M0892	ICD10	Juvenile arthritis, unspecified, elbow
M08921	ICD10	Juvenile arthritis, unspecified, right elbow
M08922	ICD10	Juvenile arthritis, unspecified, left elbow
M08929	ICD10	Juvenile arthritis, unspecified, unspecified elbow
M0893	ICD10	Juvenile arthritis, unspecified, wrist
M08931	ICD10	Juvenile arthritis, unspecified, right wrist
M08932	ICD10	Juvenile arthritis, unspecified, left wrist
M08939	ICD10	Juvenile arthritis, unspecified, unspecified wrist
M0894	ICD10	Juvenile arthritis, unspecified, hand
M08941	ICD10	Juvenile arthritis, unspecified, right hand
M08942	ICD10	Juvenile arthritis, unspecified, left hand
M08949	ICD10	Juvenile arthritis, unspecified, unspecified hand
M0895	ICD10	Juvenile arthritis, unspecified, hip
M08951	ICD10	Juvenile arthritis, unspecified, right hip
M08952	ICD10	Juvenile arthritis, unspecified, left hip
M08959	ICD10	Juvenile arthritis, unspecified, unspecified hip
M0896	ICD10	Juvenile arthritis, unspecified, knee
M08961	ICD10	Juvenile arthritis, unspecified, right knee
M08962	ICD10	Juvenile arthritis, unspecified, left knee
M08969	ICD10	Juvenile arthritis, unspecified, unspecified knee
M0897	ICD10	Juvenile arthritis, unspecified, ankle and foot
M08971	ICD10	Juvenile arthritis, unspecified, right ankle, and foot
M08972	ICD10	Juvenile arthritis, unspecified, left ankle and foot
M08979	ICD10	Juvenile arthritis, unspecified, unspecified ankle, and foot
M0898	ICD10	Juvenile arthritis, unspecified, vertebrae
M0899	ICD10	Juvenile arthritis, unspecified, multiple sites
M120	ICD10	Chronic postrheumatic arthropathy [Jaccoud’s arthropathy]
M1200	ICD10	Chronic postrheumatic arthropathy, unspecified site
M1201	ICD10	Chronic postrheumatic arthropathy [Jaccoud’s arthropathy], shoulder
M12011	ICD10	Chronic postrheumatic arthropathy [Jaccoud’s arthropathy], right shoulder
M12012	ICD10	Chronic postrheumatic arthropathy [Jaccoud’s arthropathy], left shoulder
M12019	ICD10	Chronic postrheumatic arthropathy, unspecified shoulder
M1202	ICD10	Chronic postrheumatic arthropathy [Jaccoud’s arthropathy], elbow
M12021	ICD10	Chronic postrheumatic arthropathy [Jaccoud’s arthropathy], right elbow

DIAG_FMT	CODE_TYPE	DIAG_DESC
M12022	ICD10	Chronic postrheumatic arthropathy [Jaccoud’s arthropathy], left elbow
M12029	ICD10	Chronic postrheumatic arthropathy, unspecified elbow
M1203	ICD10	Chronic postrheumatic arthropathy [Jaccoud’s arthropathy], wrist
M12031	ICD10	Chronic postrheumatic arthropathy [Jaccoud’s arthropathy], right wrist
M12032	ICD10	Chronic postrheumatic arthropathy [Jaccoud’s arthropathy], left wrist
M12039	ICD10	Chronic postrheumatic arthropathy, unspecified wrist
M1204	ICD10	Chronic postrheumatic arthropathy [Jaccoud’s arthropathy], hand
M12041	ICD10	Chronic postrheumatic arthropathy [Jaccoud’s arthropathy], right hand
M12042	ICD10	Chronic postrheumatic arthropathy [Jaccoud’s arthropathy], left hand
M12049	ICD10	Chronic postrheumatic arthropathy, unspecified hand
M1205	ICD10	Chronic postrheumatic arthropathy [Jaccoud’s arthropathy], hip
M12051	ICD10	Chronic postrheumatic arthropathy [Jaccoud’s arthropathy], right hip
M12052	ICD10	Chronic postrheumatic arthropathy [Jaccoud’s arthropathy], left hip
M12059	ICD10	Chronic postrheumatic arthropathy [Jaccoud’s arthropathy], unspecified hip
M1206	ICD10	Chronic postrheumatic arthropathy [Jaccoud’s arthropathy], knee
M12061	ICD10	Chronic postrheumatic arthropathy [Jaccoud’s arthropathy], right knee
M12062	ICD10	Chronic postrheumatic arthropathy [Jaccoud’s arthropathy], left knee
M12069	ICD10	Chronic postrheumatic arthropathy, unspecified knee
M1207	ICD10	Chronic postrheumatic arthropathy [Jaccoud’s arthropathy], ankle and foot
M12071	ICD10	Chronic postrheumatic arthropathy, right ankle and foot
M12072	ICD10	Chronic postrheumatic arthropathy, left ankle and foot
M12079	ICD10	Chronic postrheumatic arthropathy, unsp ankle and foot
M1208	ICD10	Chronic postrheumatic arthropathy, other specified site
M1209	ICD10	Chronic postrheumatic arthropathy [Jaccoud’s arthropathy], multiple sites
M123	ICD10	Palindromic rheumatism
M1230	ICD10	Palindromic rheumatism, unspecified site
M1231	ICD10	Palindromic rheumatism, shoulder
M12311	ICD10	Palindromic rheumatism, right shoulder
M12312	ICD10	Palindromic rheumatism, left shoulder
M12319	ICD10	Palindromic rheumatism, unspecified shoulder
M1232	ICD10	Palindromic rheumatism, elbow
M12321	ICD10	Palindromic rheumatism, right elbow
M12322	ICD10	Palindromic rheumatism, left elbow
M12329	ICD10	Palindromic rheumatism, unspecified elbow
M1233	ICD10	Palindromic rheumatism, wrist
M12331	ICD10	Palindromic rheumatism, right wrist
M12332	ICD10	Palindromic rheumatism, left wrist
M12339	ICD10	Palindromic rheumatism, unspecified wrist
M1234	ICD10	Palindromic rheumatism, hand
M12341	ICD10	Palindromic rheumatism, right hand
M12342	ICD10	Palindromic rheumatism, left hand
M12349	ICD10	Palindromic rheumatism, unspecified hand
M1235	ICD10	Palindromic rheumatism, hip
M12351	ICD10	Palindromic rheumatism, right hip
M12352	ICD10	Palindromic rheumatism, left hip
M12359	ICD10	Palindromic rheumatism, unspecified hip

DIAG_FMT	CODE_TYPE	DIAG_DESC
M1236	ICD10	Palindromic rheumatism, knee
M12361	ICD10	Palindromic rheumatism, right knee
M12362	ICD10	Palindromic rheumatism, left knee
M12369	ICD10	Palindromic rheumatism, unspecified knee
M1237	ICD10	Palindromic rheumatism, ankle, and foot
M12371	ICD10	Palindromic rheumatism, right ankle, and foot
M12372	ICD10	Palindromic rheumatism, left ankle and foot
M12379	ICD10	Palindromic rheumatism, unspecified ankle, and foot
M1238	ICD10	Palindromic rheumatism, other specified site
M1239	ICD10	Palindromic rheumatism, multiple sites
M30	ICD10	Polyarteritis nodosa and related conditions
M300	ICD10	Polyarteritis nodosa
M301	ICD10	Polyarteritis with lung involvement [Churg-Strauss]
M302	ICD10	Juvenile polyarteritis
M303	ICD10	Mucocutaneous lymph node syndrome [Kawasaki]
M308	ICD10	Other conditions related to polyarteritis nodosa
M310	ICD10	Hypersensitivity angiitis
M311	ICD10	Thrombotic microangiopathy
M312	ICD10	Lethal midline granuloma
M313	ICD10	Wegener's granulomatosis
M3130	ICD10	Wegener's granulomatosis without renal involvement
M3131	ICD10	Wegener's granulomatosis with renal involvement
M32	ICD10	Systemic lupus erythematosus (SLE)
M320	ICD10	Drug-induced systemic lupus erythematosus
M321	ICD10	Systemic lupus erythematosus w organ or system involvement
M3210	ICD10	Systemic lupus erythematosus, organ, or system involv unsp
M3211	ICD10	Endocarditis in systemic lupus erythematosus
M3212	ICD10	Pericarditis in systemic lupus erythematosus
M3213	ICD10	Lung involvement in systemic lupus erythematosus
M3214	ICD10	Glomerular disease in systemic lupus erythematosus
M3215	ICD10	Tubulo-interstitial neuropath in sys lupus erythematosus
M3219	ICD10	Oth organ or system involv in systemic lupus erythematosus
M328	ICD10	Other forms of systemic lupus erythematosus
M329	ICD10	Systemic lupus erythematosus, unspecified
M33	ICD10	Dermatopolymyositis
M330	ICD10	Juvenile dermatomyositis
M3300	ICD10	Juvenile dermatomyositis, organ involvement unspecified
M3301	ICD10	Juvenile dermatomyositis with respiratory involvement
M3302	ICD10	Juvenile dermatomyositis with myopathy
M3309	ICD10	Juvenile dermatomyositis with other organ involvement
M331	ICD10	Other dermatomyositis
M3310	ICD10	Other dermatomyositis, organ involvement unspecified
M3311	ICD10	Other dermatomyositis with respiratory involvement
M3312	ICD10	Other dermatomyositis with myopathy
M3319	ICD10	Other dermatomyositis with other organ involvement
M332	ICD10	Polymyositis

DIAG_FMT	CODE_TYPE	DIAG_DESC
M3320	ICD10	Polymyositis, organ involvement unspecified
M3321	ICD10	Polymyositis with respiratory involvement
M3322	ICD10	Polymyositis with myopathy
M3329	ICD10	Polymyositis with other organ involvement
M339	ICD10	Dermatopolymyositis, unspecified
M3390	ICD10	Dermatopolymyositis, unsp, organ involvement unspecified
M3391	ICD10	Dermatopolymyositis, unsp with respiratory involvement
M3392	ICD10	Dermatopolymyositis, unspecified with myopathy
M3399	ICD10	Dermatopolymyositis, unsp with other organ involvement
M34	ICD10	Systemic sclerosis [scleroderma]
M340	ICD10	Progressive systemic sclerosis
M341	ICD10	CR(E)ST syndrome
M342	ICD10	Systemic sclerosis induced by drug and chemical
M348	ICD10	Other forms of systemic sclerosis
M3481	ICD10	Systemic sclerosis with lung involvement
M3482	ICD10	Systemic sclerosis with myopathy
M3483	ICD10	Systemic sclerosis with polyneuropathy
M3489	ICD10	Other systemic sclerosis
M349	ICD10	Systemic sclerosis, unspecified
M35	ICD10	Other systemic involvement of connective tissue
M350	ICD10	Sjogren syndrome
M3500	ICD10	Sjogren syndrome, unspecified
M3501	ICD10	Sjogren syndrome with keratoconjunctivitis
M3502	ICD10	Sjogren syndrome with lung involvement
M3503	ICD10	Sjogren syndrome with myopathy
M3504	ICD10	Sjogren syndrome with tubulo-interstitial nephropathy
M3509	ICD10	Sjogren syndrome with other organ involvement
M351	ICD10	Other overlap syndromes
M352	ICD10	Behcet's disease
M353	ICD10	Polymyalgia rheumatica
M354	ICD10	Diffuse (eosinophilic) fasciitis
M355	ICD10	Multifocal fibrosclerosis
M356	ICD10	Relapsing panniculitis [Weber-Christian]
M357	ICD10	Hypermobility syndrome
M358	ICD10	Other specified systemic involvement of connective tissue
M359	ICD10	Systemic involvement of connective tissue, unspecified
M45	ICD10	Ankylosing spondylitis
M450	ICD10	Ankylosing spondylitis of multiple sites in spine
M451	ICD10	Ankylosing spondylitis of occipito-atlanto-axial region
M452	ICD10	Ankylosing spondylitis of cervical region
M453	ICD10	Ankylosing spondylitis of cervicothoracic region
M454	ICD10	Ankylosing spondylitis of thoracic region
M455	ICD10	Ankylosing spondylitis of thoracolumbar region
M456	ICD10	Ankylosing spondylitis lumbar region
M457	ICD10	Ankylosing spondylitis of lumbosacral region
M458	ICD10	Ankylosing spondylitis sacral and sacrococcygeal region

DIAG_FMT	CODE_TYPE	DIAG_DESC
M459	ICD10	Ankylosing spondylitis of unspecified sites in spine
M461	ICD10	Sacroiliitis, not elsewhere classified
M468	ICD10	Other specified inflammatory spondylopathies
M4680	ICD10	Oth inflammatory spondylopathies, site unspecified
M4681	ICD10	Oth inflammatory spondylopathies, occipt-atlan-ax region
M4682	ICD10	Oth inflammatory spondylopathies, cervical region
M4683	ICD10	Oth inflammatory spondylopathies, cervicothoracic region
M4684	ICD10	Oth inflammatory spondylopathies, thoracic region
M4685	ICD10	Oth inflammatory spondylopathies, thoracolumbar region
M4686	ICD10	Other specified inflammatory spondylopathies, lumbar region
M4687	ICD10	Oth inflammatory spondylopathies, lumbosacral region
M4688	ICD10	Oth inflammatory spondylopathies, sacr/sacrocygl region
M4689	ICD10	Oth inflammatory spondylopathies, multiple sites in spine
M469	ICD10	Unspecified inflammatory spondylopathy
M4690	ICD10	Unspecified inflammatory spondylopathy, site unspecified
M4691	ICD10	Unsp inflammatory spondylopathy, occipt-atlan-ax region
M4692	ICD10	Unspecified inflammatory spondylopathy, cervical region
M4693	ICD10	Unsp inflammatory spondylopathy, cervicothoracic region
M4694	ICD10	Unspecified inflammatory spondylopathy, thoracic region
M4695	ICD10	Unspecified inflammatory spondylopathy, thoracolumbar region
M4696	ICD10	Unspecified inflammatory spondylopathy, lumbar region
M4697	ICD10	Unspecified inflammatory spondylopathy, lumbosacral region
M4698	ICD10	Unsp inflammatory spondylopathy, sacr/sacrocygl region
M4699	ICD10	Unsp inflammatory spondylopathy, multiple sites in spine
M941	ICD10	Relapsing polychondritis
M9410	ICD10	Relapsing polychondritis, multiple sites
M9411	ICD10	Relapsing polychondritis, shoulder region
M9412	ICD10	Relapsing polychondritis, upper arm
M9413	ICD10	Relapsing polychondritis, forearm
M9414	ICD10	Relapsing polychondritis, hand
M9415	ICD10	Relapsing polychondritis, pelvic region and thigh
M9416	ICD10	Relapsing polychondritis, lower leg
M9417	ICD10	Relapsing polychondritis, ankle and foot
M9418	ICD10	Relapsing polychondritis, other site
M9419	ICD10	Relapsing polychondritis, site unspecified
O00	ICD10	Pregnancy with abortive outcome
O01	ICD10	Pregnancy with abortive outcome
O02	ICD10	Pregnancy with abortive outcome
O03	ICD10	Pregnancy with abortive outcome
O04	ICD10	Pregnancy with abortive outcome
O07	ICD10	Pregnancy with abortive outcome
O08	ICD10	Pregnancy with abortive outcome
O09	ICD10	Supervision of high-risk pregnancy
O10	ICD10	Edema, proteinuria and hypertensive disorders in pregnancy, childbirth, and the puerperium

DIAG_FMT	CODE_TYPE	DIAG_DESC
O11	ICD10	Edema, proteinuria and hypertensive disorders in pregnancy, childbirth, and the puerperium
O12	ICD10	Edema, proteinuria and hypertensive disorders in pregnancy, childbirth, and the puerperium
O13	ICD10	Edema, proteinuria and hypertensive disorders in pregnancy, childbirth, and the puerperium
O14	ICD10	Edema, proteinuria and hypertensive disorders in pregnancy, childbirth, and the puerperium
O15	ICD10	Edema, proteinuria and hypertensive disorders in pregnancy, childbirth, and the puerperium
O16	ICD10	Edema, proteinuria and hypertensive disorders in pregnancy, childbirth, and the puerperium
O20	ICD10	Other maternal disorders predominantly related to pregnancy
O21	ICD10	Other maternal disorders predominantly related to pregnancy
O22	ICD10	Other maternal disorders predominantly related to pregnancy
O23	ICD10	Other maternal disorders predominantly related to pregnancy
O24	ICD10	Other maternal disorders predominantly related to pregnancy
O25	ICD10	Other maternal disorders predominantly related to pregnancy
O26	ICD10	Other maternal disorders predominantly related to pregnancy
O28	ICD10	Other maternal disorders predominantly related to pregnancy
O29	ICD10	Other maternal disorders predominantly related to pregnancy
O30	ICD10	Maternal care related to the fetus and amniotic cavity and possible delivery problems
O31	ICD10	Maternal care related to the fetus and amniotic cavity and possible delivery problems
O32	ICD10	Maternal care related to the fetus and amniotic cavity and possible delivery problems
O33	ICD10	Maternal care related to the fetus and amniotic cavity and possible delivery problems
O34	ICD10	Maternal care related to the fetus and amniotic cavity and possible delivery problems
O35	ICD10	Maternal care related to the fetus and amniotic cavity and possible delivery problems
O36	ICD10	Maternal care related to the fetus and amniotic cavity and possible delivery problems
O40	ICD10	Maternal care related to the fetus and amniotic cavity and possible delivery problems
O41	ICD10	Maternal care related to the fetus and amniotic cavity and possible delivery problems
O42	ICD10	Maternal care related to the fetus and amniotic cavity and possible delivery problems
O43	ICD10	Maternal care related to the fetus and amniotic cavity and possible delivery problems
O44	ICD10	Maternal care related to the fetus and amniotic cavity and possible delivery problems
O45	ICD10	Maternal care related to the fetus and amniotic cavity and possible delivery problems
O46	ICD10	Maternal care related to the fetus and amniotic cavity and possible delivery problems
O47	ICD10	Maternal care related to the fetus and amniotic cavity and possible delivery problems

DIAG_FMT	CODE_TYPE	DIAG_DESC
O48	ICD10	Maternal care related to the fetus and amniotic cavity and possible delivery problems
O60	ICD10	Complications of labor and delivery
O61	ICD10	Complications of labor and delivery
O62	ICD10	Complications of labor and delivery
O63	ICD10	Complications of labor and delivery
O64	ICD10	Complications of labor and delivery
O65	ICD10	Complications of labor and delivery
O66	ICD10	Complications of labor and delivery
O67	ICD10	Complications of labor and delivery
O68	ICD10	Complications of labor and delivery
O69	ICD10	Complications of labor and delivery
O70	ICD10	Complications of labor and delivery
O71	ICD10	Complications of labor and delivery
O72	ICD10	Complications of labor and delivery
O73	ICD10	Complications of labor and delivery
O74	ICD10	Complications of labor and delivery
O75	ICD10	Complications of labor and delivery
O76	ICD10	Complications of labor and delivery
O77	ICD10	Complications of labor and delivery
O80	ICD10	Encounter for delivery
O82	ICD10	Encounter for delivery
O85	ICD10	Complications predominantly related to the puerperium
O86	ICD10	Complications predominantly related to the puerperium
O87	ICD10	Complications predominantly related to the puerperium
O88	ICD10	Complications predominantly related to the puerperium
O89	ICD10	Complications predominantly related to the puerperium
O90	ICD10	Complications predominantly related to the puerperium
O91	ICD10	Complications predominantly related to the puerperium
O92	ICD10	Complications predominantly related to the puerperium
O94	ICD10	Other obstetric conditions, not elsewhere classified
O98	ICD10	Other obstetric conditions, not elsewhere classified
O99	ICD10	Other obstetric conditions, not elsewhere classified
O9A	ICD10	Other obstetric conditions, not elsewhere classified
V22	ICD9	Normal pregnancy
V23	ICD9	Supervision of high-risk pregnancy
V24	ICD9	Postpartum care and examination
Z33	ICD10	Pregnant state
Z34	ICD10	Encounter for supervision of normal pregnancy
Z36	ICD10	Encounter for antenatal screening of mother
Z3A	ICD10	Weeks of gestation
70583	ICD9	Hidradenitis suppurativa
L732	ICD10	Hidradenitis suppurativa
3632	ICD9	Other and unspecified forms of chorioretinitis and retinochoroiditis
36011	ICD9	Sympathetic uveitis
36320	ICD9	Chorioretinitis, unspecified

DIAG_FMT	CODE_TYPE	DIAG_DESC
36404	ICD9	Secondary noninfectious iridocyclitis
0915	ICD9	Uveitis due to secondary syphilis
09150	ICD9	Syphilitic uveitis NOS
09151	ICD9	Syphilit chorioretinitis
09152	ICD9	Syphilitic iridocyclitis
3642	ICD9	Certain types of iridocyclitis
3643	ICD9	Unspecified iridocyclitis
A1854	ICD10	Tuberculous iridocyclitis (Anterior uveitis due to tuberculosis)
A5143	ICD10	Secondary syphilitic uveitis
B0051	ICD10	Herpesviral iridocyclitis (due to varicella zoster virus)
B0232	ICD10	Zoster iridocyclitis (due to herpes simplex virus)
B5809	ICD10	Other toxoplasma oculopathy (Toxoplasma uveitis)
H20011	ICD10	Primary iridocyclitis, right eye
H20012	ICD10	Primary iridocyclitis, left eye
H20013	ICD10	Primary iridocyclitis, bilateral eye
H20019	ICD10	Primary iridocyclitis, unspecified eye
H20021	ICD10	Recurrent acute iridocyclitis, right eye
H20022	ICD10	Recurrent acute iridocyclitis, left eye
H20023	ICD10	Recurrent acute iridocyclitis, bilateral eye
H20029	ICD10	Recurrent acute iridocyclitis, unspecified eye
H20031	ICD10	Secondary infectious iridocyclitis, right eye
H20032	ICD10	Secondary infectious iridocyclitis, left eye
H20033	ICD10	Secondary infectious iridocyclitis, bilateral eye
H20039	ICD10	Secondary infectious iridocyclitis, unspecified eye
H20041	ICD10	Secondary noninfectious iridocyclitis, right eye
H20042	ICD10	Secondary noninfectious iridocyclitis, left eye
H20043	ICD10	Secondary noninfectious iridocyclitis, bilateral eye
H20049	ICD10	Secondary noninfectious iridocyclitis, unspecified eye
H2011	ICD10	Chronic iridocyclitis, right eye
H2012	ICD10	Chronic iridocyclitis, left eye
H2013	ICD10	Chronic iridocyclitis, bilateral eye
H209	ICD10	Unspecified iridocyclitis
H30021	ICD10	Focal chorioretinal inflammation of posterior pole, right eye
H30022	ICD10	Focal chorioretinal inflammation of posterior pole, left eye
H30023	ICD10	Focal chorioretinal inflammation of posterior pole, bilateral eye
H30029	ICD10	Focal chorioretinal inflammation of posterior pole, unspecified eye
H30031	ICD10	Focal chorioretinal inflammation, peripheral, right eye
H30032	ICD10	Focal chorioretinal inflammation, peripheral, left eye
H30033	ICD10	Focal chorioretinal inflammation, peripheral, bilateral eye
H30039	ICD10	Focal chorioretinal inflammation, peripheral, unspecified eye
H3091	ICD10	Unspecified chorioretinal inflammation, right eye
H3092	ICD10	Unspecified chorioretinal inflammation, left eye
H3093	ICD10	Unspecified chorioretinal inflammation, bilateral eye
H4389	ICD10	Other disorders of vitreous body

DIAG_FMT	CODE_TYPE	DIAG_DESC
H44001	ICD10	Unspecified purulent endophthalmitis, right eye
H44002	ICD10	Unspecified purulent endophthalmitis, left eye
H44003	ICD10	Unspecified purulent endophthalmitis, bilateral eye
H44009	ICD10	Unspecified purulent endophthalmitis, unspecified eye
H44011	ICD10	Panophthalmitis (acute), right eye
H44012	ICD10	Panophthalmitis (acute), left eye
H44013	ICD10	Panophthalmitis (acute), bilateral eye
H44019	ICD10	Panophthalmitis (acute), unspecified eye
H44111	ICD10	Panuveitis, right eye
H44112	ICD10	Panuveitis, left eye
H44113	ICD10	Panuveitis, bilateral eye
H44119	ICD10	Panuveitis, unspecified eye
H4413	ICD10	Sympathetic uveitis
H44131	ICD10	Sympathetic uveitis right eye
H44131	ICD10	Sympathetic uveitis, right eye
H44132	ICD10	Sympathetic uveitis left eye
H44132	ICD10	Sympathetic uveitis, left eye
H44133	ICD10	Sympathetic uveitis bilateral
H44133	ICD10	Sympathetic uveitis, bilateral eye
H44139	ICD10	Sympathetic uveitis unspecified eye
H44139	ICD10	Sympathetic uveitis, unspecified

US Geographic Regions

US Region	Division	State
1. Northeast	1. New England	CT MA ME NH RI VT
	2. Mid-Atlantic	NJ NY PA
2. North Central	3. East North Central	IL IN MI OH WI
	4. West North Central	IA KS MN MO ND NE SD
3. South	5. South Atlantic	DC DE FL GA MD NC SC VA WV
	6. East South Central	AL KY MS TN
	7. West South Central	AR LA OK TX
4. West	8. Mountain	AZ CO ID MT NM NV UT WY
	9. Pacific	AK CA HI OR WA

Baseline Comorbidities

Condition	ICD9	ICD10
Anemia	280','285','64820','64821','64822','64823','64824'	D500', 'D508', 'D509', 'D62', 'D630', 'D631', 'D638', 'D640', 'D641', 'D642', 'D643', 'D644', 'D6481', 'D6489', 'D649', 'O99019', 'O99011', 'O99012', 'O99013', 'O9902', 'O9903', 'O9081'
Anxiety	'30000','30002'	F34','F40','F41','F42','F44','F45','F48','F68','F99','R45','F419','F411'
Atherosclerosis	440', '443'	I70'

Condition	ICD9	ICD10
Celiac disease	579'	K90'
Cholelithiasis	99741'	K8080', 'K8081', 'K9186'
Chronic pain	33829','3384'	G8929','G894'
Depression	2960','2961','2962','2963','2964','2965','3004','309','311'	F204','F30','F31','F32','F33','F34','F39','F3011','F3012','F3013','F302','F341','F412','F432'
Diabetes	250', '2500','2501','2502','2503','2507'	E10','E11','E12','E13','E14'
Fatigue	78071','78079'	R5382','R5383'
Fistula	5651'	K603','K604','K605'
Hyperlipidemia	2722', '2724'	E782', 'E7849', 'E785'
Hypertension	3482', '4010', '4011', '4019', '4160', '4372', '5723', '7600', '36042', '36211', '36504', '402', '403', '404', '405', '4593'	G932', 'H3503', 'H4005', 'I10', 'I110', 'I119', 'I120', 'I129', 'I130', 'I1310', 'I1311', 'I132', 'I150', 'I151', 'I152', 'I158', 'I159', 'I160', 'I161', 'I169', 'I270', 'I2720', 'I2721', 'I2722', 'I2723', 'I2724', 'I2729', 'I674', 'I873', 'I973', 'K766'
Obesity	2780','27800','27801','27802','27803'	E65','E66','E6601','E6609','E661','E662','E663','E668','E68','E669','E678','E68'
Venous thromboembolism	453'	I81','I82'
